# Supplementary material for: Prevalence and determinants of non-communicable diseases risk factors among reproductive-aged women: Findings from a nationwide survey in Bangladesh
Source: PLoS One. 2023 Jun 9;18(6):e0273128. doi: 10.1371/journal.pone.0273128 (PMC10256164; doi:10.1371/journal.pone.0273128)
Supplement: Attachment — Submitted filename: PONE-D-22-21467_R1_reviewer.pdf [file pone.0273128.s004.pdf]

PLOS ONE

Prevalence and determinants of non-communicable diseases risk factors among reproductive-aged women of Bangladesh: Findings from Bangladesh Demographic Health Survey 2017-2018

--Manuscript Draft--

**Manuscript Number:**	PONE-D-22-21467R1
**Article Type:**	Research Article
**Full Title:**	Prevalence and determinants of non-communicable diseases risk factors among reproductive-aged women of Bangladesh: Findings from Bangladesh Demographic Health Survey 2017-2018
**Short Title:**	Prevalence and determinants of non-communicable diseases risk factors among reproductive-aged women
**Corresponding Author:**	Saifur Rahman Chowdhury, MPH<br>North South University<br>Dhaka, BANGLADESH
**Keywords:**	Non-communicable diseases; risk factors; reproductive-aged women; BDHS; Bangladesh
**Abstract:**	Introduction<br>Knowing the risk factors like hypertension, overweight/obesity, and smoking status among women of reproductive age could allow the development of an effective strategy for reducing the burden of non-noncommunicable diseases (NCDs). We sought to determine the prevalence and determinants of NCDs risk factors among Bangladeshi women of reproductive age.<br>Methods<br>This study utilized the Bangladesh Demographic and Health Survey (BDHS) data from 2017-2018 and analyzed 5,624 women of reproductive age (age 18-49 years). This nationally representative cross-sectional survey utilized a stratified, two-stage sample of households. Mixed-effects Poisson regression models were fitted to find the adjusted prevalence ratio for smoking, overweight and obesity, and hypertension.<br>Results<br>The average age of 5,624 participants was 31 years (SD=9.07). The prevalence of smoking, overweight/obesity, and hypertension was 9.55%, 31.57%, and 20.27%, respectively. More than one-third of the participants (34.55%) had one NCDs risk factor, and 12.51% of participants had two NCDs risk factors. Women between 40–49 years had more NCDs risk factors than 18–29 years aged women (APR: 2.44; 95% CI: 2.22-2.68). Women with no education (APR: 1.15; 95% CI: 1.00-1.33), married (APR: 2.32; 95% CI: 1.78–3.04), and widowed/divorced (APR: 2.14; 95% CI: 1.59–2.89) were more prevalent in NCDs risk factors. Individuals in the Barishal (APR: 1.44; 95% CI: 1.28–1.63) division were living with higher risk factors for NCDs than those in the Dhaka division. Women who belonged to the richest (APR: 1.82; 95% CI: 1.60–2.07) wealth quintile were more likely to have the risk factors of NCDs.<br>Conclusions<br>This study revealed that older women, currently married and widowed/divorced women, women from the wealthiest socio-economic group, and women with a lower level of education were more likely to have NCDs risk factors. Furthermore, there was a significant relationship between the geographical division and NCDs risk factors. To reduce the future prevalence of NCDs, it is necessary to implement effective prevention and control programs that target women with a higher risk of having the disease.
**Order of Authors:**	Saifur Rahman Chowdhury, MPH
	Md Nazrul Islam, MPH
	Tasbeen Akhtar Sheekha, MPH
	Shirmin Bintay Kader, MPH
	Ahmed Hossain, PhD

Powered by Editorial Manager® and ProduXion Manager® from Aries Systems Corporation

Response to Reviewers:	Response to reviewers files attached
Additional Information:	
Question	Response
**Financial Disclosure**<br><br>Enter a financial disclosure statement that describes the sources of funding for the work included in this submission. Review the submission guidelines for detailed requirements. View published research articles from *PLOS ONE* for specific examples.<br><br>This statement is required for submission and **will appear in the published article** if the submission is accepted. Please make sure it is accurate.<br><br>**Unfunded studies**<br>Enter: *The author(s) received no specific funding for this work.*<br><br>**Funded studies**<br>Enter a statement with the following details:<br>• Initials of the authors who received each award<br>• Grant numbers awarded to each author<br>• The full name of each funder<br>• URL of each funder website<br>• Did the sponsors or funders play any role in the study design, data collection and analysis, decision to publish, or preparation of the manuscript?<br>• **NO** - Include this sentence at the end of your statement: *The funders had no role in study design, data collection and analysis, decision to publish, or preparation of the manuscript.*<br>• **YES** - Specify the role(s) played.<br><br>* typeset	The author(s) received no specific funding for this work.
**Competing Interests**<br><br>Use the instructions below to enter a competing interest statement for this submission. On behalf of all authors, disclose any competing interests that could be perceived to bias this work—acknowledging all financial support and any other relevant financial or non-financial competing interests.	The authors have declared that no competing interests exist.

Powered by Editorial Manager® and ProduXion Manager® from Aries Systems Corporation

This statement is required for submission and **will appear in the published article** if the submission is accepted. Please make sure it is accurate and that any funding sources listed in your Funding Information later in the submission form are also declared in your Financial Disclosure statement.<br><br>View published research articles from *PLOS ONE* for specific examples.<br><br>**NO authors have competing interests**<br><br>Enter: *The authors have declared that no competing interests exist.*<br><br>**Authors with competing interests**<br><br>Enter competing interest details beginning with this statement:<br><br>*I have read the journal's policy and the authors of this manuscript have the following competing interests: [insert competing interests here]*<br><br>* typeset	
**Ethics Statement**<br><br>Enter an ethics statement for this submission. This statement is required if the study involved:<br><br>• Human participants<br>• Human specimens or tissue<br>• Vertebrate animals or cephalopods<br>• Vertebrate embryos or tissues<br>• Field research<br><br>Write "N/A" if the submission does not require an ethics statement.<br><br>General guidance is provided below. Consult the submission guidelines for detailed instructions. **Make sure that all**	This study used secondary data from the country representative survey (BDHS 2017-2018). The study protocol for BDHS was approved by the ICF (international institutional review board), and the data is publicly available (http://dhsprogram.com/data/available-datasets.cfm). Therefore, no further ethical approval was necessary for this study. However, we received authorization from the DHS to use the datasets. Informed consent was obtained from each participant of the survey<br>before enrolling in the survey by using the Introduction and Consent form of the survey. It was also explained that the information would be kept strictly confidential and not be shared with anyone except the survey team members.

Powered by Editorial Manager® and ProduXion Manager® from Aries Systems Corporation

**information entered here is included in the Methods section of the manuscript.**<br><br>**Format for specific study types**<br><br>**Human Subject Research (involving human participants and/or tissue)**<br>• Give the name of the institutional review board or ethics committee that approved the study<br>• Include the approval number and/or a statement indicating approval of this research<br>• Indicate the form of consent obtained (written/oral) or the reason that consent was not obtained (e.g. the data were analyzed anonymously)<br><br>**Animal Research (involving vertebrate animals, embryos or tissues)**<br>• Provide the name of the Institutional Animal Care and Use Committee (IACUC) or other relevant ethics board that reviewed the study protocol, and indicate whether they approved this research or granted a formal waiver of ethical approval<br>• Include an approval number if one was obtained<br>• If the study involved *non-human primates*, add *additional details* about animal welfare and steps taken to ameliorate suffering<br>• If anesthesia, euthanasia, or any kind of animal sacrifice is part of the study, include briefly which substances and/or methods were applied<br><br>**Field Research**<br><br>Include the following details if this study involves the collection of plant, animal, or other materials from a natural setting:<br>• Field permit number<br>• Name of the institution or relevant body that granted permission	
**Data Availability**<br><br>Authors are required to make all data underlying the findings described fully available, without restriction, and from the time of publication. PLOS allows rare exceptions to address legal and ethical	Yes - all data are fully available without restriction

concerns. See the PLOS Data Policy and FAQ for detailed information.<br><br>A Data Availability Statement describing where the data can be found is required at submission. Your answers to this question constitute the Data Availability Statement and **will be published in the article**, if accepted.<br><br>**Important:** Stating 'data available on request from the author' is not sufficient. If your data are only available upon request, select 'No' for the first question and explain your exceptional situation in the text box.<br><br>Do the authors confirm that all data underlying the findings described in their manuscript are fully available without restriction?	
**Describe where the data may be found in full sentences. If you are copying our sample text, replace any instances of XXX with the appropriate details.**<br><br>• If the data are **held or will be held in a public repository**, include URLs, accession numbers or DOIs. If this information will only be available after acceptance, indicate this by ticking the box below. For example: *All XXX files are available from the XXX database (accession number(s) XXX, XXX.*).<br>• If the data are all contained **within the manuscript and/or Supporting Information files**, enter the following: *All relevant data are within the manuscript and its Supporting Information files.*<br>• If neither of these applies but you are able to provide **details of access elsewhere**, with or without limitations, please do so. For example:<br><br>*Data cannot be shared publicly because of [XXX]. Data are available from the XXX Institutional Data Access / Ethics Committee (contact via XXX) for researchers who meet the criteria for access to confidential data.*	All relevant data are within the manuscript and its Supporting Information files.

The data underlying the results presented in the study are available from (include the name of the third party and contact information or URL).<br>• This text is appropriate if the data are owned by a third party and authors do not have permission to share the data.<br><br>* typeset	
Additional data availability information:	

Powered by Editorial Manager® and ProduXion Manager® from Aries Systems Corporation

Manuscript
Click here to access/download;Manuscript;Manuscript-10-10-2022_Updated_Clean.docx

Research Article

Study Title:

Prevalence and determinants of non-communicable diseases risk factors among reproductive-aged women of Bangladesh: Findings from Bangladesh Demographic Health Survey 2017-2018

Short Title:

Prevalence and determinants of non-communicable diseases risk factors among reproductive-aged women of Bangladesh

Authors' Name:

Saifur Rahman Chowdhury[1,*], Md Nazrul Islam[1], Tasbeen Akhtar Sheekha[2], Shirmin Bintay Kader[2], Ahmed Hossain[1,3]

Authors' Affiliations:

[1] Department of Public Health, North South University, Dhaka-1229, Bangladesh
[2] Department of Community Health and Epidemiology, College of Medicine, University of Saskatchewan, Saskatoon, SK, S7N 5E5, Canada
[3] Health Services Administration, College of Health Sciences, University of Sharjah, Sharjah, United Arab Emirates

*Corresponding Author:

Name : Saifur Rahman Chowdhury
Email : chowdhury.saifurrahman@northsouth.edu
Address : Department Department of Public Health, North South University, Dhaka-1229, Bangladesh
ORCID : http://orcid.org/0000-0003-4361-0792

Authors' Email:

Saifur Rahman Chowdhury : chowdhury.saifurrahman@northsouth.edu
Md Nazrul Islam : nazrul.i@usask.ca
Tasbeen Akhtar Sheekha : uno401@mail.usask.ca
Shirmin Bintay Kader : shirmin.kader@usask.ca
Ahmed Hossain : ahmed.hossain@northsouth.edu

Abstract

Introduction

Knowing the risk factors like hypertension, overweight/obesity, and smoking status among women of reproductive age could allow the development of an effective strategy for reducing the burden of non-noncommunicable diseases (NCDs). We sought to determine the prevalence and determinants of NCDs risk factors among Bangladeshi women of reproductive age.

Methods

This study utilized the Bangladesh Demographic and Health Survey (BDHS) data from 2017-2018 and analyzed 5,624 women of reproductive age (age 18-49 years). This nationally representative cross-sectional survey utilized a stratified, two-stage sample of households. Mixed-effects Poisson regression models were fitted to find the adjusted prevalence ratio for smoking, overweight and obesity, and hypertension.

Results

The average age of 5,624 participants was 31 years (SD=9.07). The prevalence of smoking, overweight/obesity, and hypertension was 9.[illegible]%, 31.57%, and 20.27%, respectively. More than one-third of the participants (34.55%) had one NCDs risk factor, and 12.51% of participants had two NCDs risk factors. Women between 40–49 years had more NCDs risk factors than 18–29 years aged women (APR: 2.44; 95% CI: 2.22-2.68). Women with no education (APR: 1.15; 95% CI: 1.00-1.33), married (APR: 2.32; 95% CI: 1.78–3.04), and widowed/divorced (APR: 2.14; 95% CI: 1.59–2.89) were more prevalent in NCDs risk factors. Individuals in the Barishal (APR: 1.44; 95% CI: 1.28–1.63) division were living with higher risk factors for NCDs than those in the Dhaka division. Women who belonged to the richest (APR: 1.82; 95% CI: 1.60–2.07) wealth quintile were more likely to have the risk factors of NCDs.

Conclusions

This study revealed that older women, currently married and widowed/divorced women, women from the wealthiest socio-economic group, and women with a lower level of education were more likely to have NCDs risk factors. Furthermore, there was a significant relationship between the geographical division and NCDs risk factors. To reduce the future prevalence of NCDs, it is necessary to implement effective prevention and control programs that target women with a higher risk of having the disease.

Keywords: non-communicable diseases; risk factors; reproductive-aged women; BDHS; Bangladesh.

Introduction

The major cause of global death and disability is non-communicable diseases (NCDs) [1,2]. The most common NCDs are cardiovascular diseases (CVD), cancer, chronic respiratory diseases, and diabetes [2]. In 2022, World Health Organization (WHO) reported that each year 17 million people die from an NCDs before age 70; an estimated 86% of these premature deaths occur in low- and middle-income countries (LMICs) , and over 80% of these deaths occur due to the above-mentioned NCDs [2].

In Bangladesh, NCDs are significant cause of mortality, responsible for 572,600 deaths, a 67% of total deaths in 2016 [3]. The prevalence of NCDs has increased over the last twenty years, and it is predicted to increase more as Bangladesh is passing through a growing stage of epidemiological transition [1,4–6]. In the year 2000, NCDs were responsible for 43% of all deaths in Bangladesh [7]. This number grew to 59% in 2010 and then reached an alarming 70% in 2019 [7]. . Bangladesh surfaced as a lower-middle-income country with an experience of rapid growth in economy and urbanization in the previous decades [8]. As a result of this substantial development, there is a large number of people are leading a more sedentary lifestyle influenced by the change in dietary habits, increased supply and demand for unhealthy processed food, and physical inactivity followed by irregularities in mealtimes, smoking, and alcohol consumption [6,8–10]. Moreover, there are metabolic risk factors that contribute to the development of NCDs. The most common metabolic risk factors are obesity, high blood sugar, high blood pressure, and increased cholesterol [5]. There is an increased chance of developing NCDs when two or more of these factors combine, referred to as clustering of risk factors [11]. Consequently, a considerable proportion of the population aged 18 years and above suffered from overweight (29.4%) [12], hypertension (26.2%) [13], and diabetes (9.2%) [14] in Bangladesh. Moreover, in two meta-analyses that included the studies up to April 2017, the weighted pooled prevalence of

CVD was estimated at 5% [6], and the prevalence of metabolic syndrome and high fasting glucose were 30.0% and 28%, respectively [5].

Women are at an increased risk of developing NCDs as they mostly experience a combination of multiple behavioral and metabolic risk factors [15,16]. Globally, NCDs are responsible for 65% of premature deaths in women, with the majority of deaths occurring in LMICs [17]. According to WHO, 94% of all maternal deaths occur in LMICs, and significant causes of these deaths are pre-existing medical conditions and risk factors [18]. Evidence showed that between 1992 and 2015, the prevalence of diabetes had a threefold to fourfold increase, with a prediction to reach around 24% in men and 33.5% in women by 2030, with higher odds of multimorbidity than men [19]. The reason behind this is that women having low socio-economic conditions than men causing gender inequality which affects health outcomes [20]. The estimated global prevalence of hypertension was 24.1% for men and 20.1% for women in 2015 [21]. Studies predict a 13% increase in the prevalence of hypertension in women by 2025 [22]. Women’s fetal health and reproductive health become severely affected by the NCDs risk factors [23,24]. For example, hypertension in pregnancy increases menstrual problems, complications in pre- and post-natal periods, and maternal mortality [25]. Moreover, previous research showed that women were more likely to be overweight in urban areas because of rapid urbanization, whereas they were more likely to be underweight in rural areas because of inadequate provision of healthy food [26]. Lack of proper nutrition may lead to serious metabolic changes contributing to the development of heart disease, hypertension, and diabetes among them as well as among their future generation [9]. In addition, obesity is related to polycystic ovary syndrome (PCOS) [27], and hypertension is the common cause of higher maternal mortality and stillbirth [25]. However, women are less likely to be identified or treated as they demonstrate fewer signs and symptoms of NCDs, for instance, CVD, compared to men and get a lack of attention in case of disease prevention strategies [28].

Although Bangladesh has been successful in achieving several Sustainable Development Goals (SDG), the government and many international organizations are still working to decrease the burden of NCDs, as these are considered a challenge in the field of public health. It is evident that to prevent and control NCDs, the socio-demographic determinants of major NCDs risk factors and their clustering should be explored and understood in a systematic way. There were many studies that focused on the prevalence and risk factors of NCDs [1,4–6,10,11], in addition to diabetes, hypertension [1,13,14], overweight and obesity [12]. Though a large number of studies were nationally representative studies and few studies used earlier data or focused on a specific geographic region, studies on women of reproductive age addressing the overall social determinants and prevalence of risk factors of NCDs were very rare. This study, therefore, aimed to investigate the prevalence of risk factors of NCDs and determine their socio-demographic distributions among Bangladeshi reproductive-aged women.

Materials and Methods

Study population and data source

This study used the data from the latest Bangladesh Demographic and Health Survey (BDHS) 2017-2018 to explore the prevalence of NCDs risk factors among reproductive-aged women. The BDHS is a nationally representative cross-sectional survey and covers the entire population residing in non-institutional dwelling units in the country. The BDHS survey was performed using a stratified, two-stage household sample. In the first step, 675 enumeration areas (EAs) (250 in urban areas and 425 in rural areas) were picked with a probability proportional to the size of the EA. In the second round of sampling, an average of 30 households per EA were gathered independently from urban and rural areas and from each of the eight divisions in order to obtain statistically reliable estimates of key demographic and health factors for the entire country. The 2017-18 BDHS used six types of questionnaires: (1) the Household Questionnaire, (2) the Woman's Questionnaire (completed by ever-married women aged 15-49), (3) the Biomarker

Questionnaire, (4) two Verbal Autopsy Questionnaires to collect data on causes of death among children under age 5, (5) the Community Questionnaire, and (6) the Fieldworker Questionnaire. The first three questionnaires were based on the model questionnaires developed for the DHS-7 Program, adapted to the situation and needs in Bangladesh and taking into account the content of the instruments employed in prior BDHS surveys. The questionnaires were developed in English and then translated into and printed in Bangla. Back translations were conducted by people not involved with the Bangla translations. Detailed survey sampling and the data collection procedure have been published in the BDHS survey report [29]. On the survey, 20,250 households were selected, interviews were completed in 19,457 households with an overall 96.5% household response rate, and information was recorded from 89,819 household members [29]. Finally, this study only included 5,624 reproductive-aged women (age 18-49 years) who had their blood pressure, height and weight, and smoking status recorded (**Fig. 1**).

Fig 1. Schematic representation of the sampling procedure of the Bangladesh Demographic and Health Survey, 2017–2018

Outcome variables

The outcome variables for this research were the three risk factors for non-communicable diseases (NCDs): smoking, overweight and obesity, and hypertension. These three selected NCDs risk factors are available in the BDHS 2017-2018 survey data set.

Smoking: In the BDHS survey, participants' current smoking status was recorded as a dichotomous response.

Overweight and obesity: Height and weight measures were collected for respondents in all of the households sampled. Weight measurements were obtained using an electronic scale (model number SECA 878U) with a digital screen, and height measurements were carried out with a

measuring board (ShorrBoard). The health technician and one female technician were deployed to take both measurements. Women who were pregnant on the day of the survey visit or had given birth during the preceding two months were excluded. Body mass index (BMI) was calculated by dividing weight in kilograms by height in meters squared (kg/m^2). The overweight was considered for the individual with BMI between 25.0 and 29.9 kg/m^2, and the obese was BMI greater than or equal to 30.0 kg/m^2 [29].

Hypertension: Blood pressure was measured for all participants aged 18 and above in the subsample of one-fourth of the households. All participants who qualified for blood pressure measures were contacted and instructed on the procedure. Those who gave their consent were measured for their blood pressure. The automatic device includes separate cuffs BP monitor was used to measure blood pressure. Three blood pressure measurements were taken at intervals of approximately 10 minutes. Typically, the average of the second and third measurements was utilized to reflect the blood pressure results of respondents. Individuals were diagnosed having hypertension if they had an average systolic blood pressure level of 140 mmHg or above, or diastolic blood pressure level of 90 mmHg or above, or they were taking antihypertensive medication at the time of the survey [30,31].

Explanatory variables

This study considered the socio-demographic covariates related to smoking, overweight/obesity, and hypertension. These included the respondents' ages (18–29, 30–39, and 40–49 years), education (no education, primary, secondary, and higher-level), marital status (married, never married, and widowed/divorced), residence (rural, urban), geographical areas (Barishal, Chattogram, Dhaka, Khulna, Mymensingh, Rajshahi, Rangpur, and Sylhet divisions), occupational status (not working, service, agriculture/self-employed, and manual), wealth index (poorest, poorer, middle, richer and richest). The wealth index of the households was calculated

by the principal component analysis. The scores were given based on available consumer goods in the household (television, bicycle, car, etc.), and household characteristics (source of drinking water, sanitation facilities, flooring materials, etc.). Then the national wealth quintiles were compiled by assigning the household score to each usual household member, ranking each person in the household population by her/his score, and then dividing the distribution into five equal categories, each comprising 20% of the population [29].

Data analysis

The socio-demographic characteristics of the respondents were reported in mean and weighted percentages. The crude prevalence of smoking, overweight and obesity, and hypertension were determined after accounting for the complex survey design and survey sampling weights. Differences between categorical variables were tested using the chi-square tests. We applied mixed-effects Poisson regression models with robust variance to identify factors linked with NCDs risk factors and reported the results as adjusted prevalence ratio (APR) with a 95% confidence interval (CI). We used this model since the odds ratio estimated using logistic regression from a cross-sectional study may significantly overestimate relative risk when the outcome is common [32,33]. Secondly, in the case of convergence failure with the log-binomial model, Poisson regression with a robust variance performs better in estimating the prevalence ratio from a cross-sectional study [34]. Finally, the number of risk factors present within each participant (from 0 to 3) was counted to assess the clustering of risk factors and analyzed using the mixed-effects Poisson regression. All statistical tests were two-tailed, and a p-value of <0.05 was regarded as statistically significant. Statistical software STATA-16 (Stata Corp LP, College Station, TX, USA) was used to conduct the analysis. Since the BDHS survey was based on the two-stage stratified cluster sampling technique, recommended sample weights provided by the BDHS 2017-18 were used for the analyses [29]. So, to account for the complex survey design, we considered the sample weights, primary sampling units, and strata using “SVY” command of STATA.

Ethical considerations

This study used secondary data from the country representative survey (BDHS 2017-2018). The study protocol for BDHS was approved by the ICF (international institutional review board), and the data is publicly available (http://dhsprogram.com/data/available-datasets.cfm). Therefore, no further ethical approval was necessary for this study. However, we received authorization from the DHS to use the datasets. Informed consent was obtained from each participant of the survey before enrolling in the survey by using the Introduction and Consent form of the survey. It was also explained that the information would be kept strictly confidential and not be shared with anyone except the survey team members.

Results

Socio-demographic characteristics of study participants

The socio-demographic characteristics of the study participants (n=5,624) are shown in **Table 1**. The mean age of research participants was 31 years (SD=9.07). Approximately half (48.26%) of them were between 18 and 29 years old. In terms of educational attainment, 17.78% of the participants did not have any formal education. On the other hand, only 14.67% of respondents had education above the secondary level. In this study, 88.22% of respondents were married. More than two-thirds (71.84%) of the participants resided in rural areas. However, about one-quarter (24.44%) of the participants came from the Dhaka division, which is the capital of Bangladesh. The richest comprised 22.02% of the total participants based on the wealth index. Surprisingly, the proportions of participants in between the poorest and richer quintiles were almost identical. More than half (54.38%) of the participants were not involved in any formal work.

Table 1. Socio-demographic characteristics of participants

Prevalence of non-communicable diseases risk factors among reproductive-aged women

Table 2 depicts the prevalence of risk factors for non-communicable diseases (NCDs) among reproductive-aged women. The prevalence of smoking was 9.55% among the study participants. Smoking was more prevalent among the age group 40-49 years (19.26%), women with no educational status (19.69%), Sylhet division residents (21.69%), participants with the poorest wealth index (14.04%), and the agriculture/self-employed women (14.49%) (p <0.001). Compared to other risk factors, overweight and obesity were profound among the participants (31.57%). About 40.26% of the participants between 30-39 years were either overweight or obese (p <0.001). Likewise, overweight and obesity were more prevalent among married (33.36%), urban residents (40.18%), Chattogram division residents (36.26%), the richest (52.31%), and service holders (35.18%) (p <0.001). In this study, 20.27% of women were suffering from hypertension. Women aged 40 to 49 had the highest prevalence of hypertension (39.21%) (p <0.001). In addition, the prevalence of hypertension was higher among women who were uneducated (29.07%), and divorced/widowed (25.70%) (p <0.001).

Table 2. Prevalence of non-communicable diseases risk factors among reproductive-aged women

Furthermore, the research determined the prevalence of NCDs risk factors by the number of factors across different age groups (**Fig. 2**). More than one-third of the participants had one NCDs risk factor, and 12.51% of participants had two NCDs risk factors.

Fig. 2. Prevalence of number of non-communicable diseases risk factors among reproductive-aged women

Association between socio-demographic characteristics and non-communicable diseases risks factors

According to **Table 3**, the mixed-effects Poisson regression model revealed the adjusted association between socio-demographic characteristics and non-communicable diseases (NCDs) risk factors. The participants' age was strongly associated with the risk factors for NCDs. Compared to participants aged 18-29 years, those aged 30-39 years (APR: 1.83; 95% CI: 1.45-2.30) and 40-49 years (APR: 2.65; 95% CI: 2.07-3.38) were more likely to be smokers. The prevalence ratio for overweight and obesity in the age group 30-39 years (APR: 1.71; 95% CI: 1.55-1.89) and 40-49 years (APR: 1.77; 95% CI: 1.57-1.98) were significantly higher than the age group18-29 years. Similarly, the prevalence of hypertension was significantly higher in the 30-39 years (APR: 2.58; 95% CI: 2.18-3.05) and 40-49 years (APR: 3.94; 95% CI: 3.34-4.69) age groups compared to the younger age group. The prevalence ratio of smoking was almost 3 times higher among participants with no education (APR: 3.19; 95% CI: 1.85-5.49) compared to those with a higher level of education. Married women (APR: 2.71; 95% CI: 1.96-3.75) and widowed/divorced women (APR: 2.16; 95% CI: 1.49-3.15) demonstrated a higher prevalence of overweight and obesity compared to the never-married women.

For current smoking, prevalence ratio of the participants from Barishal (APR: 2.81; 95% CI: 2.04-3.86), Mymensingh (APR: 2.06; 95% CI: 1.47-2.90), and Sylhet (APR: 3.07; 95% CI: 2.26-4.16) was comparatively higher than the participants from Dhaka, the capital of Bangladesh. However, hypertension was almost 1.5 times higher among the participants from Barishal, Chattogram, and Rangpur than the participants from Dhaka. In addition, individuals with hypertension (APR: 1.51; 95% CI: 1.22-1.87), overweight and obesity (APR: 2.88; 95% CI: 2.39-3.47) were more likely to belong to the wealthiest quintile. Compared to those who were not employed, those whose occupations involved agriculture or self-employment had a higher prevalence ratio for current smoking (APR: 1.42; 95% CI: 1.18-1.71).

Table 3. Association between socio-demographic characteristics and non-communicable diseases risk factors identified from the multivariable mixed-effects Poisson regression model

Results of mean number NCDs risk factors and multivariable analysis of clustering of NCDs risk factors

We have examined the clustering of NCDs risk factors (the number of risk factors present in an individual from 0 to 3) in our sample. **Table 4** shows that women of 40–49 years were more likely to experience non-communicable diseases (NCDs) risk factors than 18–29 years aged women (ARR: 2.44; 95% CI: 2.22-2.68). Women with no education were at slightly higher risk for NCDs risk factors (ARR: 1.15; 95% CI: 1.00-1.33) compared to the women with a higher level of education. In comparison with the never-married women, married (ARR: 2.32; 95% CI: 1.78–3.04), and widowed/divorced (ARR: 2.14; 95% CI: 1.59–2.89) were more prevalent in NCDs risk factors.

Table 4. Mean number of NCDs risk factors and multivariable analysis (mixed-effects Poisson regression model) of clustering of NCDs risk factors

Individuals in the Barishal (ARR: 1.44; 95% CI: 1.28–1.63), Chattogram (ARR: 1.25; 95% CI: 1.12–1.40), and Sylhet divisions (ARR: 1.27; 95% CI: 1.12–1.43) were living with somewhat higher risk factors of NCDs compared to the individuals in the Dhaka division. Likewise, women belonged to the middle (ARR: 1.25; 95% CI: 1.11–1.41), richer (ARR: 1.44; 95% CI: 1.27–1.63), and richest wealth quintile (ARR: 1.82; 95% CI: 1.60–2.07) were more likely to have the risk factors of NCDs compared to the women in the poorest wealth quintile. The regression prevalence ratio plot also demonstrates the determinants of clustering of NCDs risk factors (**Fig. 3**).

Fig. 3. Regression prevalence ratio plot for the socio-demographic characteristics associated with clustering of NCDs risk factors identified from the mixed-effects Poisson regression model

Discussion

It is well-established that NCDs are increasing at an alarming rate [2]. Globally, the prevalence of NCDs was reported to be much higher in women in comparison to men [2]. The scenario is not different for Bangladesh as well [35]. It has been reported that in recent years, the prevalence of NCDs doubled in reproductive-aged women of LMICs [36]. So, our study attempted to investigate the prevalence and determinants of NCDs risk factors among reproductive-aged women of Bangladesh by using the most recent nationally representative survey.

We found that the smoking, overweight/obesity, and hypertension rates were 9.55%, 31.57%, and 20.27%, respectively, among reproductive-aged women. Our findings were similar to other previous study findings of Bangladesh, which also reported similar rates of NCDs risk factors [1,35,37,38]. The result of our study showed that the prevalence of smoking increased with age. The findings of our study were in line with the findings of a similar nationwide survey held in Nepal [39]. The high prevalence of smokers was reported commonly among women aged 40+ years, living in a rural area, illiterate, widowed/divorced, and with lower socio-economic status, which is similar to other studies [39–42]. A three-fold higher prevalence of smoking among aged (30-40 years), divorced, and illiterate might be a reflection of coping strategies, and smoking might be an option to overcome their loneliness [43]. Bista et al. found in their study in Nepal that the proportion of tobacco usage was more than 2 times higher among 30-40 years aged women and nearly four times higher among 40+years women in comparison to 15-29 years women [39]. The trend was similar for our study as well. This might be due to the cultural and socio-demographic similarities between Nepal and Bangladesh. Sreeramareddy et al. found similar results while comparing nationwide surveys in nine South-Asian countries, including Bangladesh and Nepal [44].

Respondents from urban areas reported being more smokers. This result is similar to the previous study conducted in Bangladesh [45]. Also, we have found that participants from Sylhet had reported more than 3 times higher smoking rates than the participants from Dhaka (capital). Similar area-level variation was reported in a previous study conducted in Bangladesh [46].

We observed an increasing trend in overweight/obesity and hypertension with increasing age and wealth index, similar to Bista et al. [39]. Though hypertension steadily increased with age, the prevalence of overweight and obesity inconsistently increased with age. Women in the 30-39 years age group were reported to be more obese (40.26%), whereas 40-49 years aged women had the highest rate of hypertension (39.21%). However, the prevalence of overweight and obesity was twice in both aforementioned age groups compared to the 18-29 years age group, which is aligned with the findings of Khanam et al. [47]. The increasing trend of hypertension with age is also evident in another nationwide study in Bangladesh [48]. We observed education level has vice versa relation with hypertension and overweight & obesity. The hypertension rate was higher in the illiterate group, whereas the obesity rate was higher in people who had secondary education. Similar findings were described by Sun K. et al., who explained that participants with lower educational backgrounds have to involve in more physically active work than educated persons, which explained their lower rate of overweight and obesity [49]. This result also can be explained by the fact that the participants from the agriculture/self-employed group had a lower prevalence of overweight and obesity in our study. Obesity and hypertension can both endanger women's life during pregnancy, so these alarming conditions need to be addressed, and policies should be implemented.

We found that married women were more vulnerable to hypertension and prone to be obese than never-married women. Findings were supported by previous studies [39,47]. Taking birth control methods might be an explanation for these findings [50,51]. Similar to other nationwide studies, we found that wealth index and geographical areas had a significant role in overweight/obesity and

hypertension [47,48]. Obesity and hypertension both significantly increased with the wealth index. This positive association with the wealth index is a clear reflection of less physical activity due to their sedentary lifestyle. Though there was no significant difference between the residential distribution for both obesity and hypertension, similar to Chowdhury et al., we found that the highest hypertensive participants were reported from Rangpur, and the lowest was from Sylhet. Poverty, malnutrition, and salt intake patterns might be responsible for this variation [52,53].

We also found that nearly 13% of the study participants reported having two risk factors, similar to Biswas et al.' findings [38]. We found that the clustering of NCDs risk factors increased with age and wealth index. The finding of our study was aligned with the trend of increasing NCDs risk factors found in previous studies from different countries, including Bangladesh [38,39]. Our study demonstrates that lower education is a potential confounder for NCDs risk factors. However, it was inconsistent with the findings from other population-based studies of Bangladesh [11,54]. A specific study population with a specific age range might be responded differently in existing studies that resulted in the distribution of NCDs risk factors among the respondents. Urbanization had no significant impact on NCDs risk factors, though, in participants from Barishal, NCDs risk factors were reported as approximately 1.5 times more than in Dhaka. Findings are similar to the research of Al-Zubayer et. al. [54].

Bangladesh has achieved the Millennium Development Goal-5 (MDG-5) [55]; however, the findings of this study demonstrate that reproductive-aged women are still in a vulnerable position. These findings indicate an alarming situation for NCDs among reproductive-aged women in Bangladesh. It might be a barrier for us to achieving Sustainable Development Goal (SDG) [56]. Pre-existing NCDs risk factors are well-established determinants for several adverse health outcomes, including maternal death [57]. Many adverse pregnancy outcomes are significantly associated with obesity, hypertension, and the smoking status of mothers [58–60]. Taking into

consideration the ages and gender of our study participants, these factors might be responsible for doubling the risk of unfavorable health conditions.

Strengths and Limitations

This is the first study in Bangladesh to investigate the prevalence and determinants of NCDs risk factors among reproductive-aged women. The major strength of our study is that it used a population-based, nationally representative data source. The BDHS covered rural and urban regions in all administrative divisions, making these findings generalizable to the country. Our mixed-effects Poisson regression corrects the overestimation of the effect size produced by conventional logistic regression employed in cross-sectional studies and increases the precision of the findings. Like the other studies, we had some limitations as well. As we used secondary data from a cross-sectional survey, we were only allowed to find the associations between the dependent and independent variables. We could not show any causal relationship. Secondly, the BDHS did not record other important clinical biomarkers (i.e., blood lipid profile, HbA1c, S. Creatinine), as well as behavioral factors (e.g., alcohol consumption, sleep duration), dietary factors (e.g., type and amount of food taken), physical activity, which are crucial risk factors for NCDs. So, these data could not be included in the analyses, which limits the strength of this study.

Conclusions

There was a high prevalence of non-communicable diseases (NCDs) risk factors among older women and who were currently married or widowed/divorced, from the wealthiest socio-economic groups, and with less education. Furthermore, the relationship between NCDs risk factors and geographic location was found to be significant. Lessening the burden of NCDs necessitates effective disease prevention and control measures for women through reducing the burden of hypertension, overweight/obesity, and smoking. If the government does not address these risk factors, women of reproductive age in Bangladesh will be at risk for poor health outcomes, and it

will be difficult to reach the sustainable development goal of reducing non-communicable diseases.

Acknowledgments

The authors thank MEASURE DHS (Demography and Health Surveys) for granting access to the BDHS 2017–2018 data.

Supporting information

The dataset of the study can be found in **S1 Data** file.

References

1. Chowdhury MZI, Rahman M, Akter T, Akhter T, Ahmed A, Shovon MA, et al. Hypertension prevalence and its trend in Bangladesh: evidence from a systematic review and meta-analysis. Clin Hypertens. 2020;26. doi:10.1186/s40885-020-00143-1
2. World Health Organization. Noncommunicable diseases. 2022 [cited 2 Oct 2022]. Available: https://www.who.int/news-room/fact-sheets/detail/noncommunicable-diseases
3. Noncommunicable Diseases Progress Monitor 2020. [cited 14 Jul 2022]. Available: https://www.who.int/publications/i/item/ncd-progress-monitor-2020
4. Barua L, Chandra Banik P, Mohammed S, Islam S, Faruque M. Application of country-specific Globorisk score to estimate next 10 years risk of cardiovascular diseases and its associated predictors among postmenopausal rural women of Bangladesh: A cross-sectional study in a primary care setting. Lifestyle Medicine. 2021;2: e32. doi:10.1002/LIM2.32
5. Ziaul M, Chowdhury I, Anik AM, Farhana Z, Bristi PD, Mamun BMA al, et al. Prevalence of metabolic syndrome in Bangladesh : a systematic review and meta- analysis of the studies. BMC Public Health. 2018;18: 1–14.

6. Chowdhury MZI, Haque MA, Farhana Z, Anik AM, Chowdhury AH, Haque SM, et al. Prevalence of cardiovascular disease among bangladeshi adult population: A systematic review and meta-analysis of the studies. Vasc Health Risk Manag. 2018;14: 165–181. doi:10.2147/VHRM.S166111
7. World Bank. Cause of death, by non-communicable diseases (% of total) - Bangladesh | Data. [cited 6 Oct 2022]. Available: https://data.worldbank.org/indicator/SH.DTH.NCOM.ZS?locations=BD
8. Chowdhury SR. Cardiovascular health and COVID-19: A Bangladesh perspective. Public Health Toxicology. 2022;2: 1–2. doi:10.18332/PHT/148141
9. Budreviciute A, Damiati S, Sabir DK, Onder K, Schuller-Goetzburg P, Plakys G, et al. Management and Prevention Strategies for Non-communicable Diseases (NCDs) and Their Risk Factors. Front Public Health. 2020;8: 788. doi:10.3389/FPUBH.2020.574111/BIBTEX
10. Biswas T, Islam MS, Linton N, Rawal LB. Socio-Economic Inequality of Chronic Non-Communicable Diseases in Bangladesh. PLoS One. 2016;11: e0167140. doi:10.1371/JOURNAL.PONE.0167140
11. Zaman MM, Bhuiyan MR, Karim MN, MoniruzZaman, Rahman MM, Akanda AW, et al. Clustering of non-communicable diseases risk factors in Bangladeshi adults: An analysis of STEPS survey 2013. BMC Public Health. 2015;15: 1–9. doi:10.1186/S12889-015-1938-4/FIGURES/3
12. Gupta R das, al Kibria GM. Prevalence and associated factors of underweight, overweight, and obesity Among Bangladeshi adults: An analysis of demographic and health survey 2017-18. Obes Med. 2021;23: 100342. doi:10.1016/J.OBMED.2021.100342
13. Khan MN, Oldroyd JC, Chowdhury EK, Hossain MB, Rana J, Renzetti S, et al. Prevalence, awareness, treatment, and control of hypertension in Bangladesh: Findings from National

Demographic and Health Survey, 2017–2018. The Journal of Clinical Hypertension. 2021;23: 1830–1842. doi:10.1111/JCH.14363

14. Hossain MB, Khan MN, Oldroyd JC, Rana J, Magliago DJ, Chowdhury EK, et al. Prevalence of, and risk factors for, diabetes and prediabetes in Bangladesh: Evidence from the national survey using a multilevel Poisson regression model with a robust variance. PLOS Global Public Health. 2022;2: e0000461. doi:10.1371/JOURNAL.PGPH.0000461

15. Khuwaja AK, Kadir MM. Gender differences and clustering pattern of behavioural risk factors for chronic non-communicable diseases: Community-based study from a developing country. Chronic Illn. 2010;6: 163–170. doi:10.1177/1742395309352255

16. Gluckman PD, Hanson MA, Bateson P, Beedle AS, Law CM, Bhutta ZA, et al. Towards a new developmental synthesis: adaptive developmental plasticity and human disease. The Lancet. 2009;373: 1654–1657. doi:10.1016/S0140-6736(09)60234-8

17. Yaya S, Reddy KS, Belizán JM, Pingray V. Non-communicable diseases and reproductive health in sub-Saharan Africa: Bridging the policy-implementation gaps. Reprod Health. 2020;17: 1–3. doi:10.1186/S12978-020-0857-8/METRICS

18. World Health Organization. Maternal mortality. 2019 [cited 7 Oct 2022]. Available: https://www.who.int/en/news-room/fact-sheets/detail/maternal-mortality

19. Rahman MM, Akter S, Jung J, Rahman MS, Sultana P. Trend, projection, and appropriate body mass index cut-off point for diabetes and hypertension in Bangladesh. Diabetes Res Clin Pract. 2017;126: 43–53. doi:10.1016/J.DIABRES.2017.01.008

20. Asogwa OA, Boateng D, Marzà-Florensa A, Peters S, Levitt N, van Olmen J, et al. Multimorbidity of non-communicable diseases in low-income and middle-income countries: a systematic review and meta-analysis. BMJ Open. 2022;12: 49133. doi:10.1136/bmjopen-2021-049133

21. Kim JS, Kim CG. Gender differences in hypertension treatment and control in young adults. Journal of Nursing Research. 2020;28. doi:10.1097/JNR.0000000000000359

22. Kearney PM, Whelton M, Reynolds K, Muntner P, Whelton PK, He J. Global burden of hypertension: Analysis of worldwide data. Lancet. 2005;365: 217–223. doi:10.1016/S0140-6736(05)70151-3

23. Ferrer RL, Sibai BM, Mulrow CD, Chiquette E, Stevens KR, Cornell J. Management of mild chronic hypertension during pregnancy: a review. Obstetrics and gynecology. 2000;96: 849–860. doi:10.1016/S0029-7844(00)00938-8

24. Duckitt K, Harrington D. Risk factors for pre-eclampsia at antenatal booking: systematic review of controlled studies. BMJ. 2005;330: 565–567. doi:10.1136/BMJ.38380.674340.E0

25. Ahmed I, Ali SM, Amenga-Etego S, Ariff S, Bahl R, Baqui AH, et al. Population-based rates, timing, and causes of maternal deaths, stillbirths, and neonatal deaths in south Asia and sub-Saharan Africa: a multi-country prospective cohort study. Lancet Glob Health. 2018;6: e1297–e1308. doi:10.1016/S2214-109X(18)30385-1/ATTACHMENT/365C35F3-5EFE-4F7D-9BEC-E916CB3CAE8B/MMC1.PDF

26. Zahangir M, Hasan M, Richardson A, Tabassum S. Malnutrition and non-communicable diseases among Bangladeshi women: an urban-rural comparison. Nature Publishing Group. 2017;7: 250. doi:10.1038/nutd.2017.2

27. Lim SS, Norman RJ, Davies MJ, Moran LJ. The effect of obesity on polycystic ovary syndrome: a systematic review and meta-analysis. Obesity Reviews. 2013;14: 95–109. doi:10.1111/J.1467-789X.2012.01053.X

28. DeVon HA, Ryan CJ, Ochs AL, Moshe S. Symptoms Across the Continuum of Acute Coronary Syndromes: Differences Between Women and Men. Am J Crit Care. 2008;17: 14. doi:10.4037/ajcc2008.17.1.14

29. National Institute of Population Research and Training (NIPORT) and ICF. Bangladesh Demographic and Health Survey 2017-18. Dhaka, Bangladesh, and Rockville, Maryland, USA: NIPORT and ICF [Internet]. 2020 [cited 16 Jul 2022]. Available: https://dhsprogram.com/publications/publication-FR344-DHS-Final-Reports.cfm

30. Whitworth JA. 2003 World Health Organization (WHO)/International Society of Hypertension (ISH) statement on management of hypertension. J Hypertens. 2003;21: 1983–1992. doi:10.1097/00004872-200311000-00002
31. Chobanian A v., Bakris GL, Black HR, Cushman WC, Green LA, Izzo JL, et al. The Seventh Report of the Joint National Committee on Prevention, Detection, Evaluation, and Treatment of High Blood Pressure: The JNC 7 Report. JAMA. 2003;289: 2560–2571. doi:10.1001/JAMA.289.19.2560
32. Barros AJD, Hirakata VN. Alternatives for logistic regression in cross-sectional studies: An empirical comparison of models that directly estimate the prevalence ratio. BMC Med Res Methodol. 2003;3: 1–13. doi:10.1186/1471-2288-3-21/TABLES/9
33. Tamhane AR, Westfall AO, Burkholder GA, Cutter GR. Prevalence odds ratio versus prevalence ratio: choice comes with consequences. Stat Med. 2016;35: 5730–5735. doi:10.1002/SIM.7059
34. Yelland LN, Salter AB, Ryan P. Performance of the Modified Poisson Regression Approach for Estimating Relative Risks From Clustered Prospective Data. Am J Epidemiol. 2011;174: 984–992. doi:10.1093/AJE/KWR183
35. Fottrell E, Ahmed N, Shaha SK, Jennings H, Kuddus A, Morrison J, et al. Distribution of diabetes, hypertension and non-communicable disease risk factors among adults in rural Bangladesh: a cross-sectional survey. BMJ Glob Health. 2018;3: e000787. doi:10.1136/BMJGH-2018-000787
36. Yaya S, Uthman OA, Ekholuenetale M, Bishwajit G. Socioeconomic Inequalities in the Risk Factors of Noncommunicable Diseases Among Women of Reproductive Age in Sub-saharan Africa: A Multi-Country Analysis of Survey Data. Front Public Health. 2018;6: 307. doi:10.3389/FPUBH.2018.00307

37. Flora MS, Mascie-Taylor CGN, Rahman M. Gender and locality differences in tobacco prevalence among adult Bangladeshis. Tob Control. 2009;18: 445–450. doi:10.1136/TC.2008.028142

38. Biswas T, Townsend N, Islam MS, Islam MR, das Gupta R, Das SK, et al. Association between socioeconomic status and prevalence of non-communicable diseases risk factors and comorbidities in Bangladesh: findings from a nationwide cross-sectional survey. BMJ Open. 2019;9: e025538. doi:10.1136/BMJOPEN-2018-025538

39. Bista B, Dhungana RR, Chalise B, Pandey AR. Prevalence and determinants of non-communicable diseases risk factors among reproductive aged women of Nepal: Results from Nepal Demographic Health Survey 2016. PLoS One. 2020;15: e0218840. doi:10.1371/JOURNAL.PONE.0218840

40. Palipudi KM, Sinha DN, Choudhury S, Zaman MM, Asma S, Andes L, et al. Predictors of tobacco smoking and smokeless tobacco use among adults in Bangladesh. Indian J Cancer. 2012;49: 387. doi:10.4103/0019-509X.107745

41. Nargis N, Thompson ME, Fong GT, Driezen P, Hussain AKMG, Ruthbah UH, et al. Prevalence and Patterns of Tobacco Use in Bangladesh from 2009 to 2012: Evidence from International Tobacco Control (ITC) Study. PLoS One. 2015;10. doi:10.1371/JOURNAL.PONE.0141135

42. Nath Khanal G, Khatri RB. Burden, prevention and control of tobacco consumption in Nepal: a narrative review of existing evidence. Int Health. 2020;0: 1–12. doi:10.1093/inthealth/ihaa055

43. Dyal SR, Valente TW. A Systematic Review of Loneliness and Smoking: Small Effects, Big Implications. https://doi.org/103109/1082608420151027933. 2015;50: 1697–1716. doi:10.3109/10826084.2015.1027933

44. Sreeramareddy CT, Pradhan PMS, Mir IA, Sin S. Smoking and smokeless tobacco use in nine South and Southeast Asian countries: Prevalence estimates and social determinants

from Demographic and Health Surveys. Popul Health Metr. 2014;12: 1–16. doi:10.1186/S12963-014-0022-0/TABLES/5

45. Khoorshid Riaz B, Islam MZ, Islam S, Zaman MM, Hossain MA, Rahman MM, et al. Risk factors for non-communicable diseases in Bangladesh: findings of the population-based cross-sectional national survey 2018. BMJ Open. 2020;10: 41334. doi:10.1136/bmjopen-2020-041334

46. Khan MMH, Khan A, Kraemer A, Mori M. Prevalence and correlates of smoking among urban adult men in Bangladesh: Slum versus non-slum comparison. BMC Public Health. 2009;9: 1–12. doi:10.1186/1471-2458-9-149/TABLES/5

47. Khanam M, Osuagwu UL, Sanin KI, Haque MA, Rita RS, Agho KE, et al. Underweight, Overweight and Obesity among Reproductive Bangladeshi Women: A Nationwide Survey. Nutrients. 2021;13. doi:10.3390/NU13124408

48. Sathi NJ, Islam MA, Ahmed MS, Islam SMS. Prevalence, trends and associated factors of hypertension and diabetes mellitus in Bangladesh: Evidence from BHDS 2011 and 2017–18. PLoS One. 2022;17: e0267243. doi:10.1371/JOURNAL.PONE.0267243

49. Ali NB, Dibley MJ, Islam S, Rahman MM, Raihana S, Bhuiyan SET, et al. Overweight and obesity among urban women with iron deficiency anaemia in Bangladesh. Matern Child Nutr. 2021;17. doi:10.1111/MCN.13102

50. Afshari M, Alizadeh-Navaei R, Moosazadeh M. Oral contraceptives and hypertension in women: results of the enrolment phase of Tabari Cohort Study. BMC Womens Health. 2021;21: 1–7. doi:10.1186/S12905-021-01376-4/TABLES/4

51. Linger Endalifer M, Diress G, Addisu A, Linger B. The association between combined oral contraceptive use and overweight/ obesity: a secondary data analysis of the 2016 Ethiopia Demographic and Health Survey. BMJ Open. 2020;10: 39229. doi:10.1136/bmjopen-2020-039229

52. Abdul Baker Chowdhury M, Uddin J, Haque R, Ibrahimou B. Hypertension among adults in Bangladesh: evidence from a national cross-sectional survey. 2016. doi:10.1186/s12872-016-0197-3

53. Thang NM, Popkin BM. ORIGINAL COMMUNICATION Patterns of food consumption in Vietnam: effects on socioeconomic groups during an era of economic growth. Eur J Clin Nutr. 2004;58: 145–153. doi:10.1038/sj.ejcn.1601761

54. Al-Zubayer MA, Ahammed B, Sarder MA, Kundu S, Majumder UK, Islam SMS. Double and triple burden of non-communicable diseases and its determinants among adults in Bangladesh: Evidence from a recent demographic and health survey. Int J Clin Pract. 2021;75. doi:10.1111/IJCP.14613

55. Chowdhury S, Banu LA, Chowdhury TA, Rubayet S, Khatoon S. Achieving Millennium Development Goals 4 and 5 in Bangladesh. BJOG. 2011;118: 36–46. doi:10.1111/j.1471-0528.2011.03111.x

56. Sustainable Development Goal 3: Good Health and Well-being | United Nations in Bangladesh. [cited 1 Jul 2022]. Available: https://bangladesh.un.org/en/sdgs/3

57. Storm F, Agampodi S, Eddleston M, Sørensen JB, Konradsen F, Rheinländer T. Indirect causes of maternal death. Lancet Glob Health. 2014;2: e566. doi:10.1016/S2214-109X(14)70297-9

58. Panaitescu AM, Syngelaki A, Prodan N, Akolekar R, Nicolaides KH. Chronic hypertension and adverse pregnancy outcome: a cohort study. Ultrasound Obstet Gynecol. 2017;50: 228–235. doi:10.1002/UOG.17493

59. Anderson TM, Lavista Ferres JM, You Ren S, Moon RY, Goldstein RD, Ramirez JM, et al. Maternal Smoking Before and During Pregnancy and the Risk of Sudden Unexpected Infant Death. Pediatrics. 2019;143. doi:10.1542/PEDS.2018-3325

60. Cedergren MI. Maternal morbid obesity and the risk of adverse pregnancy outcome. Obstetrics and gynecology. 2004;103: 219–224. doi:10.1097/01.AOG.0000107291.46159.00

Table 1. Socio-demographic characteristics of participants

Variables	Un-weighted count	Weighted percent/Mean (SD)
**Total**	5,624	100.00
**Mean age (year)**		31.00 (9.07)
**Age group (year)**		
18 – 29	2,689	48.26
30 – 39	1,716	30.26
40 – 49	1,219	21.48
**Educational status**		
No education	957	17.78
Primary	1,691	29.87
Secondary	2,051	37.68
Higher	925	14.67
**Marital status**		
Never married	402	6.33
Married	4,902	88.22
Widowed/divorced	320	5.45
**Residence**		
Rural	3,533	71.84
Urban	2,091	28.16
**Division**		
Barishal	590	5.47
Chattogram	827	18.57
Dhaka	799	24.44
Khulna	729	11.69
Mymensingh	589	7.50
Rajshahi	732	14.24
Rangpur	690	11.62
Sylhet	668	6.46
**Wealth index**		
Poorest	1,069	19.01
Poorer	1,051	19.50
Middle	1,081	19.90
Richer	1,091	19.56
Richest	1,332	22.02
**Occupational status***		
Not working	3,066	54.38
Services	242	4.01
Agriculture/self-employed	1,667	30.22
Manual	641	11.39

*8 cases missing

Table 2. Prevalence of non-communicable diseases risk factors among reproductive-aged women

	Current smoking		Overweight and obesity		Hypertension	
**Variables**	**n**	**Prevalence, % (95% CI)**	**n**	**Prevalence, % (95% CI)**	**n**	**Prevalence, % (95% CI)**
**Total**	5,624	9.55 (8.65-10.53)	5,603	31.57 (30.17-33.02)	5,624	20.27 (19.11-21.49)
**Age group (year)**						
18 – 29	2,689	4.28 (3.53-5.17)	2,679	22.92 (21.11-24.82)	2,689	9.03 (7.87-10.33)
30 – 39	1,716	11.06 (9.48 -12.86)	1,708	40.26 (37.63-42.94)	1,716	24.77 (22.48-27.20)
40 – 49	1,219	19.26 (16.69 -22.12)	1,216	38.82 (35.91-41.82)	1,219	39.21 (36.16-42.35)
P-value		<0.001		<0.001		<0.001
**Educational status**						
No education	957	19.69 (17.05-22.62)	952	26.13 (23.26-29.21)	957	29.07 (25.92-32.43)
Primary	1,691	12.58 (10.95-14.41)	1,688	30.75 (28.35-33.27)	1,691	22.29 (20.16-24.57)
Secondary	2,051	5.07 (4.17-6.15)	2,044	33.81 (31.42-36.29)	2,051	17.14 (15.39-19.04)
Higher	925	2.60 (1.67-4.05)	919	34.08 (30.44-37.92)	925	13.57 (11.33-16.17)
P-value		<0.001		0.001		<0.001
**Marital status**						
Never married	402	1.39 (0.53-3.57)	396	10.43 (7.47-14.38)	402	6.18 (4.01-9.40)
Married	4,902	9.71 (8.78-10.73)	4,890	33.36 (31.88-34.87)	4,902	20.95 (19.69-22.27)
Widowed/divorced	320	16.41 (2.42-21.38)	317	26.85 (21.57-32.87)	320	25.70 (20.60-31.56)
P-value		<0.001		<0.001		<0.001
**Residence**						
Rural	3,533	10.21 (9.11-11.42)	3,519	28.20 (26.53-29.94)	3,533	19.92 (18.50-21.41)
Urban	2,091	7.87 (6.45-9.57)	2,084	40.18 (37.63-42.78)	2,091	21.19 (19.21-23.31)
P-value		0.024		<0.001		0.317
**Division**						
Barishal	590	21.29 (17.65-25.45)	588	29.28 (25.13-33.80)	590	24.24 (20.43-28.50)
Chattogram	827	8.62 (6.64-11.10)	824	36.26 (32.57-40.11)	827	23.51 (20.71-26.57)
Dhaka	799	6.35 (4.77-8.41)	795	35.73 (32.44-39.16)	799	16.90 (14.37-19.78)
Khulna	729	6.83 (4.77-9.70)	728	35.45 (31.80-39.29)	729	20.47 (17.29-24.05)
Mymensingh	589	17.01 (13.72-20.89)	587	24.69 (21.03-28.75)	589	15.76 (12.94-19.06)
Rajshahi	732	5.06 (3.24-7.82)	728	28.13 (24.64-31.90)	732	20.62 (17.36-24.32)
Rangpur	690	8.92 (6.10-12.86)	689	25.20 (21.89-28.83)	690	23.36 (20.51-26.48)
Sylhet	668	21.69 (17.73-26.24)	664	24.40 (20.11-29.26)	668	18.93 (15.78-22.55)
P-value		<0.001		<0.001		0.001
**Wealth index**						
Poorest	1,069	14.04 (11.72-16.75)	1,066	16.48 (14.10-19.19)	1,069	17.35 (14.77-20.28)
Poorer	1,051	12.46 (10.43-14.81)	1,046	21.74 (19.12-24.62)	1,051	18.84 (16.44-21.48)
Middle	1,081	8.37 (6.76-10.31)	1,080	29.80 (26.82-32.97)	1,081	20.10 (17.66-22.78)
Richer	1,091	8.42 (6.80-10.39)	1,088	34.59 (31.47-37.84)	1,091	21.39 (18.82-24.21)
Richest	1,332	5.16 (3.98-6.66)	1,323	52.31 (49.24-55.37)	1,332	23.24 (20.81-25.86)
P-value		<0.001		<0.001		0.019
**Occupational status***						
Not working	3,066	7.53 (6.53-8.67)	3,052	34.35 (32.41-36.34)	3,066	20.19 (18.65-21.83)
Services	242	5.62 (3.15-9.85)	238	35.18 (28.54-42.45)	242	20.76 (15.51-27.20)
Agriculture/self-employed	1,667	14.49 (12.57-16.63)	1,667	25.97 (23.85-28.20)	1,667	22.25 (20.03-24.63)
Manual	641	7.60 (5.33-10.72)	638	32.46 (28.52-36.68)	641	15.56 (12.83-18.75)
P-value		<0.001		<0.001		0.013

*8 cases missing

Table 3. Association between socio-demographic characteristics and non-communicable diseases risk factors identified from the multivariable mixed-effects Poisson regression model

Variables	Current smoking, APR (95% CI)	Overweight and obesity, APR (95% CI)	Hypertension, APR (95% CI)
**Age group (year)**			
18 – 29	Reference	Reference	Reference
30 – 39	1.83 (1.45-2.30)***	1.71 (1.55-1.89) ***	2.58 (2.18-3.05)***
40 – 49	2.65 (2.07-3.38)***	1.77 (1.57-1.98) ***	3.94 (3.32-4.69)***
**Educational status**			
No education	3.19 (1.85-5.49)***	0.81 (0.69-0.97)*	1.33 (1.04-1.71)*
Primary	2.41 (1.43-4.04) **	0.98 (0.85-1.12)	1.27 (1.01-1.61)*
Secondary	1.45 (0.87-2.43)	1.04 (0.92-1.17)	1.23 (0.98-1.53)
Higher	Reference	Reference	Reference
**Marital status**			
Never married	Reference	Reference	Reference
Married	2.87 (1.10-7.52)*	2.71 (1.96-3.75)***	1.60 (1.02-2.51)*
Widowed/divorced	3.32 (1.22-9.03)*	2.16 (1.49-3.15)***	1.53 (0.94-2.50)
**Residence**			
Rural	Reference	Reference	Reference
Urban	1.23 (1.00-1.50)*	1.01 (0.92-1.10)	1.06 (0.94-1.21)
**Division**			
Barishal	2.81 (2.04-3.86)***	1.07 (0.91-1.26)	1.50 (1.20-1.87)***
Chattogram	1.41 (1.00-1.98)*	1.11 (0.98-1.26)	1.45 (1.20-1.77)***
Dhaka	Reference	Reference	Reference
Khulna	0.91 (0.62-1.34)	1.11 (0.97-1.28)	1.18 (0.96-1.46)
Mymensingh	2.06 (1.47-2.90)***	0.93 (0.78-1.11)	1.02 (0.79-1.31)
Rajshahi	0.61 (0.40-0.92)*	1.01 (0.87-1.18)	1.27 (1.02-1.57)*
Rangpur	1.00 (0.68-1.46)	1.03 (0.87-1.22)	1.52 (1.23-1.89)***
Sylhet	3.07 (2.26-4.16)***	0.87 (0.74-1.02)	1.19 (0.96-1.49)
**Wealth index**			
Poorest	Reference	Reference	Reference
Poorer	1.00 (0.81-1.24)	1.27 (1.05-1.54)*	1.08 (0.89-1.31)
Middle	0.79 (0.62-1.02)	1.67 (1.39-2.01)***	1.19 (0.98-1.44)
Richer	0.90 (0.68-1.17)	1.97 (1.64-2.36)***	1.36 (1.12-1.67)**
Richest	0.66 (0.46-0.94)*	2.88 (2.39-3.47)***	1.51 (1.22-1.87)***
**Occupational status**			
Not working	Reference	Reference	Reference
Services	1.12 (0.63-2.00)	0.94 (0.77-1.14)	1.04 (0.78-1.38)
Agriculture/self-employed	1.42 (1.18 -1.71)***	0.88 (0.79-0.98)*	0.91 (0.80-1.04)
Manual	0.94 (0.69-1.27)	1.00 (0.87-1.14)	0.76 (0.62-0.93)**

APR: Adjusted prevalence ratio
***Significant at p-value < 0.001.
**Significant at p-value < 0.01.
*Significant at p-value < 0.05.

Table 4. Mean number of NCDs risk factors and multivariable analysis (mixed-effects Poisson regression model) of clustering of NCDs risk factors

Variables	Mean number (95% CI)	Clustering of NCDs risk factors, APR (95% CI)
**Age group (year)**		
18 – 29	0.36 (0.34-0.39)	Reference
30 – 39	0.76 (0.72-0.80)	1.94 (1.78-2.11)***
40 – 49	0.97 (0.92-1.03)	2.44 (2.22-2.68)***
**Educational status**		
No education	0.75 (0.69-0.80)	1.15 (1.00-1.33)*
Primary	0.66 (0.61-0.70)	1.13 (1.00-1.29)*
Secondary	0.56 (0.53-0.60)	1.09 (0.97-1.23)
Higher	0.50 (0.45-0.55)	Reference
**Marital status**		
Never married	0.18 (0.13-0.23)	Reference
Married	0.64 (0.62-0.66)	2.32 (1.78-3.04)***
Widowed/divorced	0.69 (0.59-0.78)	2.14 (1.59-2.89)***
**Residence**		
Rural	0.58 (0.56-0.61)	Reference
Urban	0.69 (0.65-0.73)	1.06 (0.99-1.14)
**Division**		
Barishal	0.75 (0.68-0.82)	1.44 (1.28-1.63)***
Chattogram	0.68 (0.63-0.74)	1.25 (1.12-1.40)***
Dhaka	0.59 (0.53-0.64)	Reference
Khulna	0.63 (0.57-0.69)	1.10 (0.97-1.24)
Mymensingh	0.58 (0.51-0.64)	1.13 (0.98-1.30)
Rajshahi	0.54 (0.48-0.60)	1.02 (0.90-1.16)
Rangpur	0.58 (0.52-0.63)	1.15 (1.01-1.32)*
Sylhet	0.65 (0.58-0.72)	1.27 (1.12-1.43)***
**Wealth index**		
Poorest	0.48 (0.43-0.53)	Reference
Poorer	0.53 (0.48-0.58)	1.11 (0.99-1.26)
Middle	0.58 (0.54-0.63)	1.25 (1.11-1.41)***
Richer	0.64 (0.59-0.69)	1.44 (1.27-1.63)***
Richest	0.81 (0.76-0.85)	1.82 (1.60-2.07)***
**Occupational status**		
Not working	0.62 (0.59-0.65)	Reference
Services	0.62 (0.50-0.73)	0.99 (0.83-1.18)
Agriculture/self-employed	0.63 (0.59-0.67)	0.98 (0.90-1.06)
Manual	0.55 (0.49-0.61)	0.90 (0.80-1.01)

APR: Adjusted prevalence ratio
***Significant at p-value < 0.001.
**Significant at p-value < 0.01.
*Significant at p-value < 0.05.

Figure

Click here to access/download;Figure;Fig-1.tif

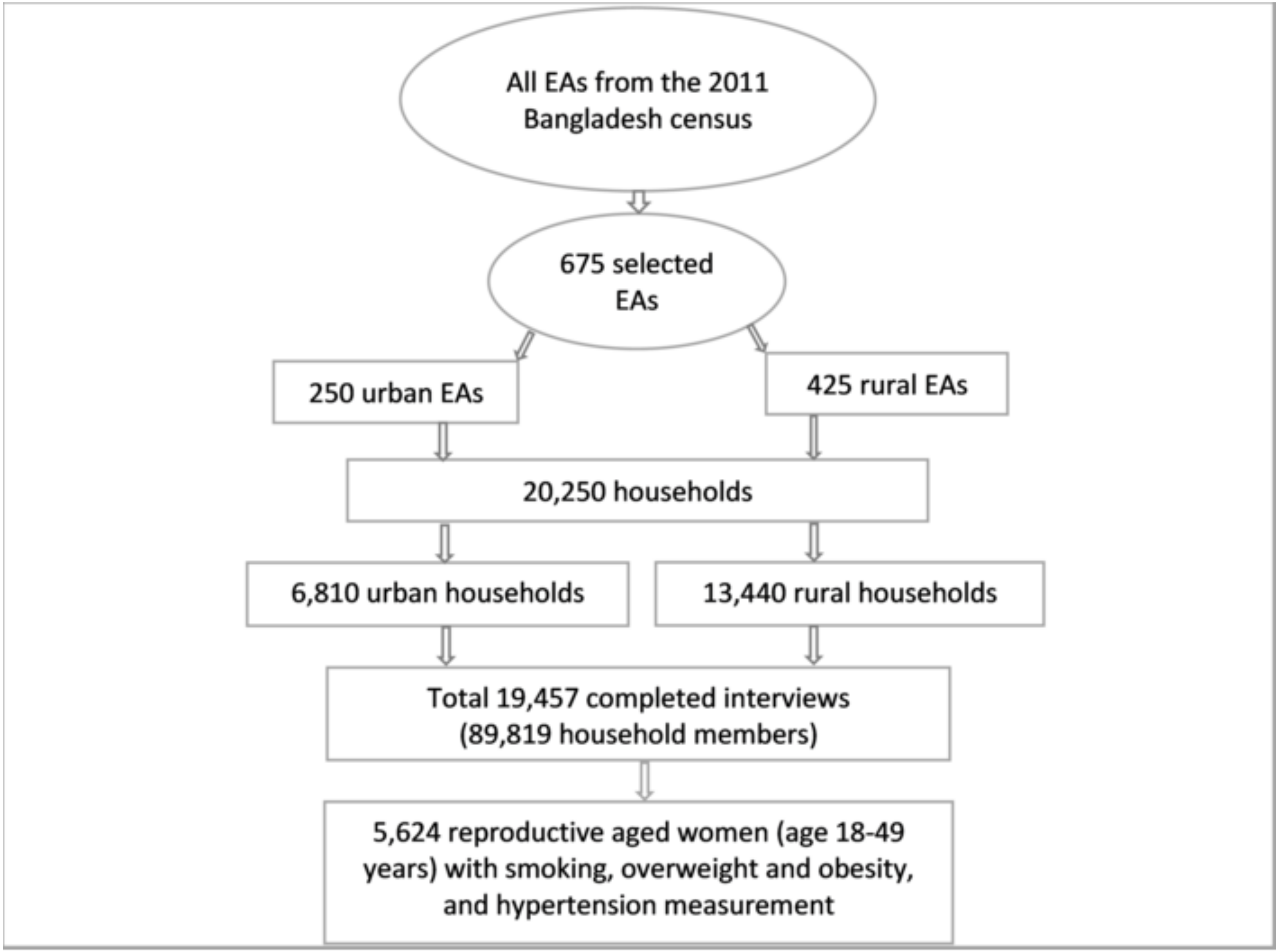

Figure
Click here to access/download;Figure;Fig-2.tif

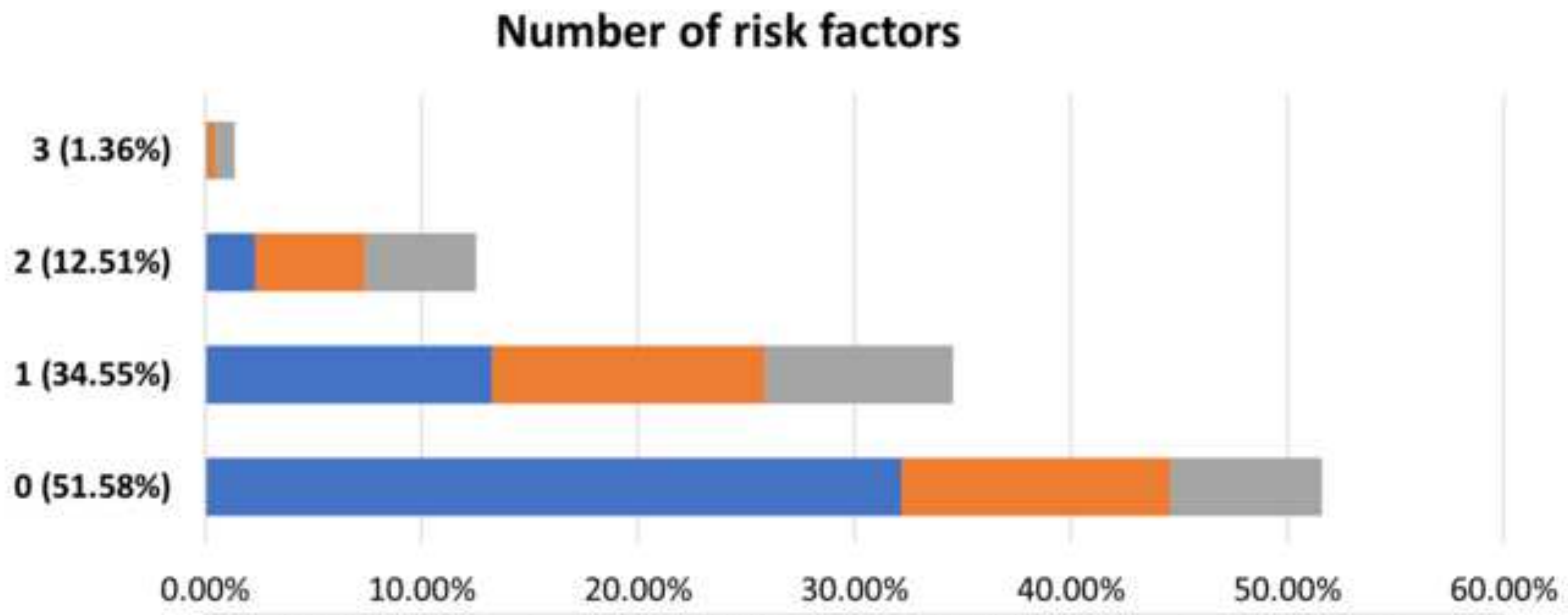

	0 (51.58%)	1 (34.55%)	2 (12.51%)	3 (1.36%)
18-29 years	32.16%	13.24%	2.32%	0.09%
30-39 years	12.42%	12.62%	5.05%	0.39%
40-49 years	7.00%	8.69%	5.14%	0.88%

Click here to access/download;Figure;Fig-3.tif

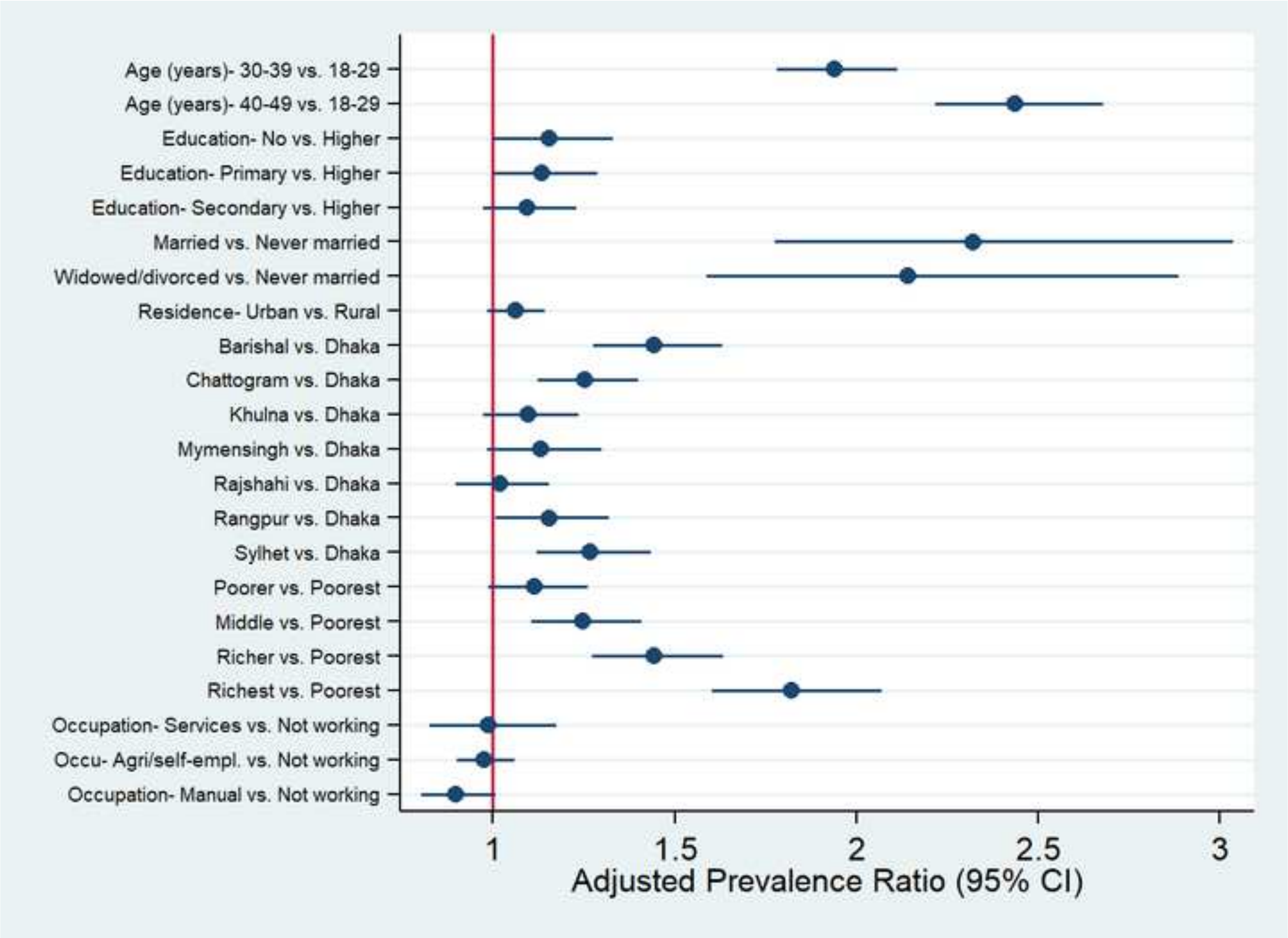

Click here to access/download
Supporting Information
S1_Data.DTA

Revised Manuscript with Track Changes

Research Article

Study Title:

Prevalence and determinants of non-communicable diseases risk factors among reproductive-aged women of Bangladesh: ~~Evidence~~Findings from Bangladesh Demographic Health Survey 2017-2018

Short Title:

Prevalence and determinants of non-communicable diseases risk factors among reproductive-aged women of Bangladesh

Authors' Name:

Saifur Rahman Chowdhury[1,*], Md Nazrul Islam[1], Tasbeen Akhtar Sheekha[2], Shirmin Bintay Kader[2], Ahmed Hossain[1,3,~~4~~]

Authors' Affiliations:

[1] Department of Public Health, North South University, Dhaka-1229, Bangladesh

[2] Department of Community Health and Epidemiology, College of Medicine, University of Saskatchewan, Saskatoon, SK, S7N 5E5, Canada

~~[3] NSU Global Health Institute (NGHI), Dhaka-1229, Bangladesh~~

[~~4~~] Health Services Administration, College of Health Sciences~~ (CHS)~~, University of Sharjah, Sharjah, United Arab Emirates ~~(UAE)~~

Formatted: Font: (Default) Arial

*Corresponding Author:

Name : Saifur Rahman Chowdhury

Email : chowdhury.saifurrahman@northsouth.edu

Address : Department Department of Public Health, North South University, Dhaka-1229, Bangladesh

ORCID : http://orcid.org/0000-0003-4361-0792

Authors' Email:

Saifur Rahman Chowdhury : chowdhury.saifurrahman@northsouth.edu

Md Nazrul Islam : nazrul.i@usask.ca

Field Code Changed

Tasbeen Akhtar Sheekha : uno401@mail.usask.ca

Shirmin Bintay Kader : shirmin.kader@usask.ca

Ahmed Hossain : ahmed.hossain@northsouth.edu

Abstract

Introduction

Knowing the risk factors like hypertension, overweight/obesity, and smoking status among women of reproductive age could allow the development of an effective strategy for reducing the burden of non-noncommunicable diseases (NCDs). We sought to determine the prevalence and determinants of NCDs risk factors among Bangladeshi women of reproductive age.

Methods

This study utilized the Bangladesh Demographic and Health Survey (BDHS) data from 2017-2018 and analyzed 5,624 women of reproductive age (age 18-49 years). This nationally representative cross-sectional survey utilized a stratified, two-stage sample of households. Mixed-effects Poisson regression models were fitted to find the adjusted prevalence ratio for smoking, overweight and obesity, and hypertension.

Results

The average age of 5,624 participants was 31 years (SD=9.07). The prevalence of smoking, overweight/obesity, and hypertension was 9.55%, 31.57%, and 20.27%, respectively. More than one-third of the participants (34.55%) had one NCDs risk factor, and 12.51% of participants had two NCDs risk factors. Women between 40–49 years had more NCDs risk factors than 18–29 years aged women (APR: 2.44; 95% CI: 2.22-2.68). Women with no education (APR: 1.15; 95% CI: 1.00-1.33), married (APR: 2.32; 95% CI: 1.78–3.04), and widowed/divorced (APR: 2.14; 95% CI: 1.59–2.89) were more prevalent in NCDs risk factors. Individuals in the Barishal (APR: 1.44; 95% CI: 1.28–1.63) division were living with higher risk factors for NCDs than those in the Dhaka division. Women who belonged to the richest (APR: 1.82; 95% CI: 1.60–2.07) wealth quintile were more likely to have the risk factors of NCDs.

Conclusions

This study revealed that older women, currently married and widowed/divorced women, women from the wealthiest socio-economic group, and women with a lower level of education were more likely to have NCDs risk factors. Furthermore, there was a significant relationship between the geographical division and NCDs risk factors. To reduce the future prevalence of NCDs, it is necessary to implement effective prevention and control programs that target women with a higher risk of having the disease.

Keywords: non-communicable diseases; risk factors; reproductive-aged women; BDHS; Bangladesh.

Introduction

The major cause of global death and disability is non-communicable diseases (NCDs) [1,2]. The most common NCDs are cardiovascular diseases (CVD), cancer, chronic respiratory diseases, and diabetes [2]. In 2022, World Health Organization (WHO) reported that each year 17 million people die from an NCDs before age 70; ~~Every year~~ an estimated 86%~~5%~~ of ~~15 million~~ these premature deaths occur ~~due to NCD~~ in low- and middle-income countries (LMICs) ~~worldwide~~ , and ~~almost~~over 80% of th~~e~~ese deaths occur due to the above-mentioned NCDs [2]. ~~Between 2012 and 2030, the number of deaths due to NCD is predicted to reach 52 million from 38 million globally.~~

In Bangladesh, NCDs are significant cause of mortality, responsible for ~~67% of total~~ 572,600 deaths, a 67% of total deaths in 2016 [3]. The prevalence of NCDs has increased over the last ~~ten~~twenty years, and it is predicted to increase more as Bangladesh is passing through a growing stage of epidemiological transition [1,4–6]. In the year 2000, NCDs were responsible for 43% of all deaths in Bangladesh [7]. This number grew to 59% in 2010 and then reached an alarming 70% in 2019 [7]. ~~Bangladesh is a lower-middle-income country having over 170 million people~~. ~~It~~ Bangladesh surfaced as a lower-middle-income country ~~developing country~~ with an experience of rapid growth in economy and urbanization in the previous decades [8]. As a result of this substantial development, there is a large number of people are leading a more sedentary lifestyle influenced by the change in dietary habits, increased supply and demand for unhealthy processed food, and physical inactivity followed by irregularities in mealtimes, smoking, and alcohol consumption [6,8–10]. Moreover, there are metabolic risk factors that contribute to the development of NCDs. The most common metabolic risk factors are obesity, high blood sugar, high blood pressure, and increased cholesterol [5]. There is an increased chance of developing NCDs when two or more of these factors combine, referred to as clustering of risk factors [11]. Consequently, a considerable proportion of the population aged 18 years and above suffered

from overweight (29.4%) [12], hypertension (26.2%) [13], and diabetes (9.2%) [14] in Bangladesh. Moreover, in two meta-analyses that included the studies up to April 2017, the weighted pooled prevalence of CVD was estimated at 5% [6], and the prevalence of metabolic syndrome and high fasting glucose were 30.0% and 28%, respectively [5].

Formatted: Font: 11 pt

Women are at an increased risk of developing NCDs as they mostly experience a combination of multiple behavioral and metabolic risk factors [15,16]. Globally, NCDs are responsible for 65% of premature deaths in women, with the majority of deaths occurring in LMICs [17]. According to WHO, 94% of all maternal deaths occur in LMICs, and significant causes of these deaths are pre-existing medical conditions and risk factors [18]. Evidence showed that between 1992 and 2015, the prevalence of diabetes ~~has~~ had a threefold to fourfold increase, with a prediction to reach around 24% in men and 33.5% in women by 2030, with higher odds of multimorbidity than men [19]. The reason behind this is that women having low socio-economic conditions than men causing gender inequality which affects health outcomes [20]. The estimated global prevalence of hypertension was 24.1% for men and 20.1% for women in 2015 [21]. Studies predict a 13% increase in the prevalence of hypertension in women by 2025 [22]. Women's ~~The~~ fetal health and ~~the~~ reproductive health ~~of women~~ become severely affected by the NCDs risk factors [23,24]. For example, hypertension in pregnancy increases menstrual problems, complications in pre- and post-natal periods, and maternal mortality [25]. Moreover, previous research showed that women were more likely to be overweight in urban areas because of rapid urbanization, whereas they were more likely to be underweight in rural areas because of inadequate provision of healthy food [26]. Lack of proper nutrition may lead to serious metabolic changes contributing to the development of heart disease, hypertension, and diabetes among them as well as among their future generation [9]. In addition, obesity is related to polycystic ovary syndrome (PCOS) [27], and hypertension is the common cause of higher maternal mortality and stillbirth [25]. However, women are less likely to be identified or treated as they demonstrate fewer signs and symptoms

of NCDs, for instance, CVD, compared to men and get a lack of attention in case of disease prevention strategies [28].

Although Bangladesh has been successful in achieving several Sustainable Development Goals (SDG), the government and many international organizations are still working to decrease the burden of NCDs, as these are considered a challenge in the field of public health. It is evident that to prevent and control NCDs, the socio-demographic determinants of major NCDs risk factors and their clustering should be explored and understood in a systematic way. There ~~a~~were many studies that focused on the prevalence and risk factors of NCDs~~s~~ [1,4–6,10,11], in addition to diabetes, hypertension [1,13,14], overweight and obesity [12]. Though a large number of studies were nationally representative studies and few studies used earlier data or focused on a specific geographic region, studies on ~~the~~ women of reproductive age addressing the overall social determinants and prevalence of risk factors of NCDs were very rare. This study, therefore, aimed to investigate the prevalence of risk factors of NCDs and determine their socio-demographic distributions among Bangladeshi reproductive-aged women.

Materials and Methods

Study population and data source

This study used the data from the latest Bangladesh Demographic and Health Survey (BDHS) 2017-2018 to explore the prevalence of ~~non-communicable disease~~NCDs risk factors among reproductive-aged women. The BDHS is a nationally representative cross-sectional survey and covers the entire population residing in non-institutional dwelling units in the country. The BDHS survey was performed using a stratified, two-stage household sample. In the first step, 675 enumeration areas (EAs) (250 in urban areas and 425 in rural areas) were picked with a probability proportional to the size of the EA. In the second round of sampling, an average of 30 households per EA were gathered independently from urban and rural areas and from each of the eight divisions in order to obtain statistically reliable estimates of key demographic and health

factors for the entire country. The 2017-18 BDHS used six types of questionnaires: (1) the Household Questionnaire, (2) the Woman's Questionnaire (completed by ever-married women aged 15-49), (3) the Biomarker Questionnaire, (4) two Verbal Autopsy Questionnaires to collect data on causes of death among children under age 5, (5) the Community Questionnaire, and (6) the Fieldworker Questionnaire. The first three questionnaires were based on the model questionnaires developed for the DHS-7 Program, adapted to the situation and needs in Bangladesh and taking into account the content of the instruments employed in prior BDHS surveys. The questionnaires were developed in English and then translated into and printed in Bangla. Back translations were conducted by people not involved with the Bangla translations. Detailed survey sampling and the data collection procedure have been published in the BDHS survey report [29]. On the survey, 20,250 households were selected, interviews were completed in 19,457 households with an overall 96.5% household response rate, and information was recorded from 89,819 household members [29]. Finally, this study only included 5,624 reproductive-aged women (age 18-49 years) who had their blood pressure, height and weight, and smoking status recorded (**Fig. 1**).

Fig 1. Schematic representation of the sampling procedure of the Bangladesh Demographic and Health Survey, 2017–2018

Outcome variables

The outcome variables for this research were the three risk factors for non-communicable diseases (NCDs)~~, which are~~: smoking, overweight and obesity, and hypertension. These three selected NCDs risk factors are available in the BDHS 2017-2018 survey data set.

Smoking: In the BDHS survey, participants' current smoking status was recorded as a dichotomous response.

Formatted: Font: Bold

Formatted: Font:

Overweight and obesity: Height and weight measures were collected for respondents in all of the households sampled. Weight measurements were obtained using an electronic scale (model number SECA 878U) with a digital screen, and height measurements were carried out with a measuring board (ShorrBoard). The health technician and one female technician were deployed to take both measurements. Women who were pregnant on the day of the survey visit or had given birth during the preceding two months were excluded. Body mass index (BMI) was calculated by dividing weight in kilograms by height in meters squared (kg/m^2). The overweight was considered for the individual with BMI between 25.0 and 29.9 kg/m^2, and the obese was BMI greater than or equal to 30.0 kg/m^2 [29].

Hypertension: Blood pressure was measured for all participants aged 18 and above in the subsample of one-fourth of the households. All participants who qualified for blood pressure measures were contacted and instructed on the procedure. Those who gave their consent were measured for their blood pressure. The automatic device includes separate cuffs BP monitor was used to ~~measuring~~ measure ~~the~~ blood pressure. Three blood pressure measurements were taken at intervals of approximately 10 minutes. Typically, the average of the second and third measurements was utilized to reflect the blood pressure results of respondents. Individuals were diagnosed having hypertension if they had an average systolic blood pressure level of 140 mmHg or above, ~~and~~or diastolic blood pressure level of 90 mmHg or above, or they were taking antihypertensive medication at the time of the survey [30,31].

Explanatory variables

This study considered the socio-demographic covariates related to smoking, overweight/obesity, and hypertension. These included the respondents' ages (18–29, 30–39, and 40–49 years), education (no education, primary, secondary, and higher-level), marital status (married, never married, and widowed/divorced), residence (rural, urban), geographical areas (Barishal,

~~Chittagong~~Chattogram, Dhaka, Khulna, Mymensingh, Rajshahi, Rangpur, and Sylhet divisions), occupational status (not working, service, agriculture/self-employed, and manual), wealth index (poorest, poorer, middle, richer and richest). The wealth index of the households was calculated by the principal component analysis. The scores were given based on available consumer goods in the household (television, bicycle, car, etc.), and household characteristics (source of drinking water, sanitation facilities, flooring materials, etc.). Then the national wealth quintiles were compiled by assigning the household score to each usual household member, ranking each person in the household population by her/his score, and then dividing the distribution into five equal categories, each comprising 20% of the population [29].

Data analysis

The socio-demographic characteristics of the respondents were reported in mean and weighted percentages. The crude prevalence of smoking, overweight and obesity, and hypertension ~~was~~ were determined after accounting for the complex survey design and survey sampling weights. Differences between categorical variables were tested using the chi-square tests. We applied mixed-effects Poisson regression models with robust variance to identify factors linked with ~~non-communicable diseases~~NCDs risk factors and reported the results as adjusted prevalence ratio (APR) with a 95% confidence interval (CI). We used this model since the odds ratio estimated using logistic regression from a cross-sectional study may significantly overestimate relative risk when the outcome is common [32,33]. Secondly, in the case of convergence failure with the log-binomial model, Poisson regression with a robust variance performs better in estimating the prevalence ratio from a cross-sectional study [34]. Finally, the number of risk factors present within each participant (from 0 to 3) was counted to assess the clustering of risk factors and analyzed using the mixed-effects Poisson regression. All statistical tests were two-tailed, and a p-value of <0.05 was regarded as statistically significant. Statistical software STATA-16 (Stata Corp LP, College Station, TX, USA) was used to conduct the analysis. Since the BDHS survey

Formatted: Don't adjust space between Latin and Asian text, Don't adjust space between Asian text and numbers

was based on the two-stage stratified cluster sampling technique, recommended sample weights provided by the BDHS 2017-18 were used for the analyses [29]. So, to account for the complex survey design, we considered the sample weights, primary sampling units, and strata using “SVY” command of STATA.

Formatted: English (Canada)

Ethical considerations

This study used secondary data from the country representative survey (BDHS 2017-2018). The study protocol for BDHS was approved by the ICF (international institutional review board), and the data is publicly available (http://dhsprogram.com/data/available-datasets.cfm). Therefore, no further ethical approval was necessary for this study. However, we received authorization from the DHS to use the datasets. Informed consent was obtained from each participant of the survey before enrolling in the survey by using the Introduction and Consent form of the survey. It was also explained that the information ~~will~~ would be kept strictly confidential and ~~will~~ not be shared with anyone except ~~with~~ the survey team members. ~~of the survey team.~~

Results

Socio-demographic characteristics of study participants

The socio-demographic characteristics of the study participants (n=5,624) are shown in **Table 1**. The mean age of research participants was 31 years (SD=9.07). Approximately half (48.26%) of them were between 18 and 29 years old. In terms of educational attainment, 17.78% of the participants did not have any formal education. On the other hand, only 14.67% of respondents had~~ve~~ education above the secondary level. In this study, 88.22% of respondents were married. More than two-thirds (71.84%) of the participants resided in rural areas. However, about one-quarter (24.44%) of the participants came from the Dhaka division, which is the capital ~~city~~ of Bangladesh. The richest comprised 22.02% of the total participants based on the wealth index.

Surprisingly, the proportions of participants in between the poorest and richer quintiles were almost identical. More than half (54.38%) of the participants were not involved in any formal work.

Table 1. Socio-demographic characteristics of participants

Prevalence of non-communicable diseases risk factors among reproductive-aged women

Table 2 depicts the prevalence of risk factors for non-communicable diseases (NCDs) among reproductive-aged women. The prevalence of smoking was 9.55% among the study participants. Smoking was more prevalent among the age group 40-49 years (19.26%), women with no educational status (19.69%), Sylhet division residents (21.69%), participants with the poorest wealth index (14.04%), and the agriculture/self-employed women (14.49%) (p <0.001). Compared to other risk factors, overweight and obesity were profound among the participants (31.57%). About 40.26% of the participants between 30-39 years were either overweight or obese (p <0.001). Likewise, overweight and obesity were more prevalent among married (33.36%), urban residents (40.18%), Chattogram division residents (36.26%), the richest (52.31%), and service holders (35.18%) (p <0.001). In this study, 20.27% of women were suffering from hypertension. Women aged 40 to 49 had the highest prevalence of hypertension (39.21%) (p <0.001). In addition, the prevalence of hypertension was higher among women who were uneducated (29.07%), and divorced/widowed (25.70%) (p <0.001).

Table 2. Prevalence of non-communicable diseases risk factors among reproductive-aged women

Furthermore, the research determined the prevalence of NCDs risk factors by the number of factors across different age groups (**Fig. 2**). More than one-third of the participants had one NCDs risk factor, and 12.51% of participants had two NCDs risk factors.

Fig. 2. Prevalence of number of non-communicable diseases risk factors among reproductive-aged women

Association between socio-demographic characteristics and non-communicable diseases risks factors

According to **Table 3**, the mixed-effects Poisson regression model revealed the adjusted association between socio-demographic characteristics and non-communicable diseases (NCDs) risk factors. The participants' age was strongly associated with the risk factors for NCDs. non-communicable diseases. Compared to participants aged 18-29 years, those aged 30-39 years (APR: 1.83; 95% CI: 1.45-2.30) and 40-49 years (APR: 2.65; 95% CI: 2.07-3.38) were more likely to be smokers. The prevalence ratio for overweight and obesity in the age group 30-39 years (APR: 1.71; 95% CI: 1.55-1.89) and 40-49 years (APR: 1.77; 95% CI: 1.57-1.98) were significantly higher than the age group18-29 years. Similarly, the prevalence of hypertension was significantly higher in the 30-39 years (APR: 2.58; 95% CI: 2.18-3.05) and 40-49 years (APR: 3.94; 95% CI: 3.34-4.69) age groups compared to the younger age group. The prevalence ratio of smoking was almost 3 times higher among participants with no education (APR: 3.19; 95% CI: 1.85-5.49) compared to those with a higher level of education. Married women (APR: 2.71; 95% CI: 1.96-3.75) and widowed/divorced women (APR: 2.16; 95% CI: 1.49-3.15) demonstrated a higher prevalence of overweight and obesity compared to the never-married women.

For current smoking, prevalence ratio of the participants from Barishal (APR: 2.81; 95% CI: 2.04-3.86), Mymensingh (APR: 2.06; 95% CI: 1.47-2.90), and Sylhet (APR: 3.07; 95% CI: 2.26-4.16) was comparatively higher than the participants from Dhaka, the capital of Bangladesh. However, hypertension was almost 1.5 times higher among the participants from Barishal, Chattogram, and Rangpur than the participants from Dhaka. In addition, individuals with hypertension (APR: 1.51; 95% CI: 1.22-1.87), overweight and obesity (APR: 2.88; 95% CI: 2.39-3.47) were more likely to belong to the wealthiest quintile. When compared to the group of peoplCompared to those who

were not employed, those whose occupations involved agriculture or self-employment had a higher prevalence ratio for current smoking (APR: 1.42; 95% CI: 1.18-1.71).

Table 3. Association between socio-demographic characteristics and non-communicable diseases risk factors identified from the multivariable mixed-effects Poisson regression model

Results of mean number NCDs risk factors and multivariable analysis of clustering of NCDs risk factors

We have examined the clustering of NCDs risk factors (the number of risk factors present in an individual from 0 to 3) in our sample. **Table 4** shows that women of 40–49 years were more likely to experience non-communicable diseases (NCDs) risk factors than 18–29 years aged women (ARR: 2.44; 95% CI: 2.22-2.68). Women with no education were at slightly higher risk for NCDs risk factors (ARR: 1.15; 95% CI: 1.00-1.33) compared to the women with a higher level of education. In comparison with the never-married women, married (ARR: 2.32; 95% CI: 1.78–3.04), and widowed/divorced (ARR: 2.14; 95% CI: 1.59–2.89) were more prevalent in NCDs risk factors.

Table 4. Mean number of NCDs risk factors and multivariable analysis (mixed-effects Poisson regression model) of clustering of NCDs risk factors

Individuals in the Barishal (ARR: 1.44; 95% CI: 1.28–1.63), Chattogram (ARR: 1.25; 95% CI: 1.12–1.40), and Sylhet divisions (ARR: 1.27; 95% CI: 1.12–1.43) ~~divisions~~ were living with somewhat higher risk factors of NCDs compared to the individuals in the Dhaka division. Likewise, women belonged to the middle (ARR: 1.25; 95% CI: 1.11–1.41), richer (ARR: 1.44; 95% CI: 1.27–1.63), and richest wealth quintile (ARR: 1.82; 95% CI: 1.60–2.07) were more likely to have the risk factors of NCDs compared to the women in the poorest wealth quintile. ~~Furthermore, t~~The

regression prevalence ratio plot also demonstrates the determinants of clustering of NCDs risk factors (**Fig. 3**).

Fig. 3. Regression prevalence ratio plot for the socio-demographic characteristics associated with clustering of NCDs risk factors identified from the mixed-effects Poisson regression model

Discussion

It is well-~~-~~established that NCDs are increasing at an alarming rate [2]. Globally, the prevalence of NCDs was reported to be much higher in women in comparison to men [2]. The scenario is not different for Bangladesh as well [35]. It has been reported that in recent years, the prevalence of NCDs doubled in reproductive-aged women of LMICs [36]. So, our study attempted to investigate the prevalence and determinants of NCDs risk factors among reproductive-aged women of Bangladesh by using the most recent nationally representative survey.

We found that the smoking, overweight/obesity, and hypertension rates were 9.55%, 31.57%, and 20.27%, respectively, among reproductive-aged women. Our findings were similar to other previous study findings of Bangladesh, ~~that~~ which also reported similar rates of NCDs risk factors [1,35,37,38]. The result of our study showed that the prevalence of smoking increased with age. The findings of our study were in line with the findings of a similar nationwide survey held in Nepal [39]. The high prevalence of smokers was reported commonly among women aged 40+ years, living in a rural area, illiterate, widowed/divorced, and with lower socio-economic status, which is similar to other studies [39–42]. ~~Similar to previous studies, a~~A three-fold higher prevalence of smoking among aged (30-40 years), divorced, and illiterate might be a reflection of coping strategies, and smoking might be an option to overcome their loneliness [43]. Bista et al. found in their study in Nepal that the proportion of tobacco usage was more than 2 times higher among 30-40 years aged women and nearly four times higher among 40+years women in comparison to 15-29 years women [39]. The trend was similar for our study as well. This might be due to the cultural

and socio-demographic similarities between Nepal and Bangladesh. Sreeramareddy et al. found similar results while comparing nationwide surveys in nine South-Asian countries, including Bangladesh and Nepal [44]. Respondents from urban areas reported being more smokers. This result is similar to the previous study conducted in Bangladesh [45]. Also, we have found that participants from Sylhet had reported more than 3 times higher smoking rates than the participants from Dhaka (capital). Similar ~~area~~ area-level variation was reported in a previous study conducted in Bangladesh [46].

We observed an increasing trend in overweight/obesity and hypertension with increasing age and wealth index, similar to Bista et al. [39]. Though hypertension steadily increased with age, the prevalence of overweight and obesity inconsistently increased with age. Women in the 30-39 years age group were reported to be more obese (40.26%), whereas 40-49 years aged women had the highest rate of hypertension (39.21%). However, the prevalence of overweight and obesity was twice in both aforementioned age groups compared to the 18-29 years age group, which is aligned with the findings of Khanam et al. [47]. The increasing trend of hypertension with age is also evident in another nationwide study in Bangladesh [48]. We observed education level has vice versa relation with hypertension and overweight & obesity. The hypertension rate was higher in the illiterate group, whereas the obesity rate was higher in people who had secondary education. Similar findings were described by Sun K. et al., who explained that participants with lower educational backgrounds have to involve in more physically active work than educated persons, which explained their lower rate of overweight and obesity [49]. This result also can be explained by the fact that the participants from the agriculture/self-employed group had a lower prevalence of overweight and obesity in our study. Obesity and hypertension ~~both can endanger women's life during pregnancy, so these alarming conditions need to be addressed and should intervene policies~~can both endanger women's life during pregnancy, so these alarming conditions need to be addressed, and policies should be implemented.

We found that married women were more vulnerable to hypertension and prone to be obese than never-married women. Findings were supported by previous studies [39,47]. Taking birth control methods might be an explanation for these findings [50,51]. Similar to other nationwide studies, we found that wealth index and geographical areas had a significant role in overweight/obesity and hypertension [47,48]. Obesity and hypertension both significantly increased with the wealth index. This positive association with the wealth index is a clear reflection of less physical activity due to their sedentary lifestyle. Though there was no significant difference between the residential distribution for both obesity and hypertension, similar to Chowdhury et al., we found that the highest hypertensive participants were reported from Rangpur, and the lowest was from Sylhet. Poverty, malnutrition, and salt intake patterns might be responsible for this variation [52,53].

We have also found that nearly 13% of the study participants population reported having two risk factors, which was similar to the findings of Biswas et al.' findings [38]. We have seen found that the clustering of NCDs risk factors increased with age and wealth index. The finding of our study was aligned with the trend of increasing NCDs risk factors found in previous studies from different countries, including Bangladesh [38,39]. Our study demonstrates that lower education is a potential confounder for NCDs risk factors. However, it was inconsistent with the findings from other population-based studies of Bangladesh [11,54]. A specific study population with a specific age range might be responded differently infrom existing studies that resulted in the distribution of NCDs risk factors among the respondents. Urbanization had no significant impact on NCDs risk factors, though, in participants from Barishal, NCDs risk factors were reported as approximately 1.5 times more than in Dhaka. Findings are similar to the research of Al-Zubayer et. al. [54].

Bangladesh has achieved the Millennium Development Goal-5 (MDG-5) [55]; however, the findings of this study demonstrate that reproductive-aged women are still in a vulnerable position. These findings indicate an alarming situation for NCDs among reproductive-aged women in Bangladesh. It might be a barrier for us to achieving Sustainable Development Goal (SDG) [56].

Pre-existing NCDs risk factors are well-established determinants for several adverse health outcomes, including maternal death [57]. Many adverse pregnancy outcomes are significantly associated with obesity, hypertension, and the smoking status of mothers [58–60]. Taking into consideration the ages and gender ~~Considering the age~~ of our study participants, these factors might be responsible for doubling the risk of unfavorable health conditions.

Strengths and Limitations

This ~~study is the first study in Bangladesh that investigated~~is the first study in Bangladesh to investigate the prevalence and determinants of NCDs risk factors among reproductive-aged women. The major strength of our study is that it used a population-based, nationally representative data source. The BDHS covered rural and urban regions in all administrative divisions, making these findings generalizable to the country. Our mixed-effects Poisson regression corrects the overestimation of the effect~~s~~ size produced by conventional logistic regression employed in cross-sectional studies and increases the precision of the findings. Like the other studies, we had some limitations as well. As we used ~~a~~ secondary data from a cross-sectional survey~~source~~, we were only allowed to find ~~out~~ the associations between the dependent and independent variables. We ~~failed~~could not ~~to~~ show any causal relationship. Secondly, the BDHS did not record other important clinical biomarkers (i.e., blood lipid profile, HbA1c, S. Creatinine), as well as behavioral factors (e.g., alcohol consumption, sleep duration), dietary factors (e.g., type and amount of food taken), physical activity, which are crucial risk factors for NCDs. So, these data could not be included in the analyses, which limits the strength of this study.

Conclusions

There was a high prevalence of ~~NCD~~non-communicable diseases (NCDs) risk factors among older women and who were currently married or widowed/divorced, from the wealthiest socio-economic groups, and with less education. Furthermore, the relationship between NCDs risk

factors and geographic location was found to be significant. Lessening the burden of NCDs ~~non-communicable diseases~~ necessitates effective disease prevention and control measures for women through reducing the burden of hypertension, overweight/obesity, and smoking. If the government does not address these risk factors, women of reproductive age in Bangladesh will be at risk for poor health outcomes, and it will be difficult to reach the sustainable development goal of reducing non-communicable diseases.

Acknowledgments

The authors thank MEASURE DHS (Demography and Health Surveys) for granting access to the BDHS 2017–2018 data.

Supporting information

~~Dataset~~ The dataset of the study can be found in **S1 Data** file.

References

1. Chowdhury MZI, Rahman M, Akter T, Akhter T, Ahmed A, Shovon MA, et al. Hypertension prevalence and its trend in Bangladesh: evidence from a systematic review and meta-analysis. Clin Hypertens. 2020;26. doi:10.1186/s40885-020-00143-1
2. World Health Organization. Noncommunicable diseases. 2022 [cited 2 Oct 2022]. Available: https://www.who.int/news-room/fact-sheets/detail/noncommunicable-diseases
3. Noncommunicable Diseases Progress Monitor 2020. [cited 14 Jul 2022]. Available: https://www.who.int/publications/i/item/ncd-progress-monitor-2020
4. Barua L, Chandra Banik P, Mohammed S, Islam S, Faruque M. Application of country-specific Globorisk score to estimate next 10 years risk of cardiovascular diseases and its associated predictors among postmenopausal rural women of Bangladesh: A cross-

sectional study in a primary care setting. Lifestyle Medicine. 2021;2: e32. doi:10.1002/LIM2.32

5. Ziaul M, Chowdhury I, Anik AM, Farhana Z, Bristi PD, Mamun BMA al, et al. Prevalence of metabolic syndrome in Bangladesh : a systematic review and meta- analysis of the studies. BMC Public Health. 2018;18: 1–14.

6. Chowdhury MZI, Haque MA, Farhana Z, Anik AM, Chowdhury AH, Haque SM, et al. Prevalence of cardiovascular disease among bangladeshi adult population: A systematic review and meta-analysis of the studies. Vasc Health Risk Manag. 2018;14: 165–181. doi:10.2147/VHRM.S166111

7. World Bank. Cause of death, by non-communicable diseases (% of total) - Bangladesh | Data. [cited 6 Oct 2022]. Available: https://data.worldbank.org/indicator/SH.DTH.NCOM.ZS?locations=BD

8. Chowdhury SR. Cardiovascular health and COVID-19: A Bangladesh perspective. Public Health Toxicology. 2022;2: 1–2. doi:10.18332/PHT/148141

9. Budreviciute A, Damiati S, Sabir DK, Onder K, Schuller-Goetzburg P, Plakys G, et al. Management and Prevention Strategies for Non-communicable Diseases (NCDs) and Their Risk Factors. Front Public Health. 2020;8: 788. doi:10.3389/FPUBH.2020.574111/BIBTEX

10. Biswas T, Islam MS, Linton N, Rawal LB. Socio-Economic Inequality of Chronic Non-Communicable Diseases in Bangladesh. PLoS One. 2016;11: e0167140. doi:10.1371/JOURNAL.PONE.0167140

11. Zaman MM, Bhuiyan MR, Karim MN, MoniruzZaman, Rahman MM, Akanda AW, et al. Clustering of non-communicable diseases risk factors in Bangladeshi adults: An analysis of STEPS survey 2013. BMC Public Health. 2015;15: 1–9. doi:10.1186/S12889-015-1938-4/FIGURES/3

12. Gupta R das, al Kibria GM. Prevalence and associated factors of underweight, overweight, and obesity Among Bangladeshi adults: An analysis of demographic and health survey 2017-18. Obes Med. 2021;23: 100342. doi:10.1016/J.OBMED.2021.100342
13. Khan MN, Oldroyd JC, Chowdhury EK, Hossain MB, Rana J, Renzetti S, et al. Prevalence, awareness, treatment, and control of hypertension in Bangladesh: Findings from National Demographic and Health Survey, 2017–2018. The Journal of Clinical Hypertension. 2021;23: 1830–1842. doi:10.1111/JCH.14363
14. Hossain MB, Khan MN, Oldroyd JC, Rana J, Magliago DJ, Chowdhury EK, et al. Prevalence of, and risk factors for, diabetes and prediabetes in Bangladesh: Evidence from the national survey using a multilevel Poisson regression model with a robust variance. PLOS Global Public Health. 2022;2: e0000461. doi:10.1371/JOURNAL.PGPH.0000461
15. Khuwaja AK, Kadir MM. Gender differences and clustering pattern of behavioural risk factors for chronic non-communicable diseases: Community-based study from a developing country. Chronic Illn. 2010;6: 163–170. doi:10.1177/1742395309352255
16. Gluckman PD, Hanson MA, Bateson P, Beedle AS, Law CM, Bhutta ZA, et al. Towards a new developmental synthesis: adaptive developmental plasticity and human disease. The Lancet. 2009;373: 1654–1657. doi:10.1016/S0140-6736(09)60234-8
17. Yaya S, Reddy KS, Belizán JM, Pingray V. Non-communicable diseases and reproductive health in sub-Saharan Africa: Bridging the policy-implementation gaps. Reprod Health. 2020;17: 1–3. doi:10.1186/S12978-020-0857-8/METRICS
18. World Health Organization. Maternal mortality. 2019 [cited 7 Oct 2022]. Available: https://www.who.int/en/news-room/fact-sheets/detail/maternal-mortality
19. Rahman MM, Akter S, Jung J, Rahman MS, Sultana P. Trend, projection, and appropriate body mass index cut-off point for diabetes and hypertension in Bangladesh. Diabetes Res Clin Pract. 2017;126: 43–53. doi:10.1016/J.DIABRES.2017.01.008

20. Asogwa OA, Boateng D, Marzà-Florensa A, Peters S, Levitt N, van Olmen J, et al. Multimorbidity of non-communicable diseases in low-income and middle-income countries: a systematic review and meta-analysis. BMJ Open. 2022;12: 49133. doi:10.1136/bmjopen-2021-049133
21. Kim JS, Kim CG. Gender differences in hypertension treatment and control in young adults. Journal of Nursing Research. 2020;28. doi:10.1097/JNR.0000000000000359
22. Kearney PM, Whelton M, Reynolds K, Muntner P, Whelton PK, He J. Global burden of hypertension: Analysis of worldwide data. Lancet. 2005;365: 217–223. doi:10.1016/S0140-6736(05)70151-3
23. Ferrer RL, Sibai BM, Mulrow CD, Chiquette E, Stevens KR, Cornell J. Management of mild chronic hypertension during pregnancy: a review. Obstetrics and gynecology. 2000;96: 849–860. doi:10.1016/S0029-7844(00)00938-8
24. Duckitt K, Harrington D. Risk factors for pre-eclampsia at antenatal booking: systematic review of controlled studies. BMJ. 2005;330: 565–567. doi:10.1136/BMJ.38380.674340.E0
25. Ahmed I, Ali SM, Amenga-Etego S, Ariff S, Bahl R, Baqui AH, et al. Population-based rates, timing, and causes of maternal deaths, stillbirths, and neonatal deaths in south Asia and sub-Saharan Africa: a multi-country prospective cohort study. Lancet Glob Health. 2018;6: e1297–e1308. doi:10.1016/S2214-109X(18)30385-1/ATTACHMENT/365C35F3-5EFE-4F7D-9BEC-E916CB3CAE8B/MMC1.PDF
26. Zahangir M, Hasan M, Richardson A, Tabassum S. Malnutrition and non-communicable diseases among Bangladeshi women: an urban-rural comparison. Nature Publishing Group. 2017;7: 250. doi:10.1038/nutd.2017.2
27. Lim SS, Norman RJ, Davies MJ, Moran LJ. The effect of obesity on polycystic ovary syndrome: a systematic review and meta-analysis. Obesity Reviews. 2013;14: 95–109. doi:10.1111/J.1467-789X.2012.01053.X

28. DeVon HA, Ryan CJ, Ochs AL, Moshe S. Symptoms Across the Continuum of Acute Coronary Syndromes: Differences Between Women and Men. Am J Crit Care. 2008;17: 14. doi:10.4037/ajcc2008.17.1.14
29. National Institute of Population Research and Training (NIPORT) and ICF. Bangladesh Demographic and Health Survey 2017-18. Dhaka, Bangladesh, and Rockville, Maryland, USA: NIPORT and ICF [Internet]. 2020 [cited 16 Jul 2022]. Available: https://dhsprogram.com/publications/publication-FR344-DHS-Final-Reports.cfm
30. Whitworth JA. 2003 World Health Organization (WHO)/International Society of Hypertension (ISH) statement on management of hypertension. J Hypertens. 2003;21: 1983–1992. doi:10.1097/00004872-200311000-00002
31. Chobanian A v., Bakris GL, Black HR, Cushman WC, Green LA, Izzo JL, et al. The Seventh Report of the Joint National Committee on Prevention, Detection, Evaluation, and Treatment of High Blood Pressure: The JNC 7 Report. JAMA. 2003;289: 2560–2571. doi:10.1001/JAMA.289.19.2560
32. Barros AJD, Hirakata VN. Alternatives for logistic regression in cross-sectional studies: An empirical comparison of models that directly estimate the prevalence ratio. BMC Med Res Methodol. 2003;3: 1–13. doi:10.1186/1471-2288-3-21/TABLES/9
33. Tamhane AR, Westfall AO, Burkholder GA, Cutter GR. Prevalence odds ratio versus prevalence ratio: choice comes with consequences. Stat Med. 2016;35: 5730–5735. doi:10.1002/SIM.7059
34. Yelland LN, Salter AB, Ryan P. Performance of the Modified Poisson Regression Approach for Estimating Relative Risks From Clustered Prospective Data. Am J Epidemiol. 2011;174: 984–992. doi:10.1093/AJE/KWR183
35. Fottrell E, Ahmed N, Shaha SK, Jennings H, Kuddus A, Morrison J, et al. Distribution of diabetes, hypertension and non-communicable disease risk factors among adults in rural

Bangladesh: a cross-sectional survey. BMJ Glob Health. 2018;3: e000787. doi:10.1136/BMJGH-2018-000787

36. Yaya S, Uthman OA, Ekholuenetale M, Bishwajit G. Socioeconomic Inequalities in the Risk Factors of Noncommunicable Diseases Among Women of Reproductive Age in Sub-saharan Africa: A Multi-Country Analysis of Survey Data. Front Public Health. 2018;6: 307. doi:10.3389/FPUBH.2018.00307

37. Flora MS, Mascie-Taylor CGN, Rahman M. Gender and locality differences in tobacco prevalence among adult Bangladeshis. Tob Control. 2009;18: 445–450. doi:10.1136/TC.2008.028142

38. Biswas T, Townsend N, Islam MS, Islam MR, das Gupta R, Das SK, et al. Association between socioeconomic status and prevalence of non-communicable diseases risk factors and comorbidities in Bangladesh: findings from a nationwide cross-sectional survey. BMJ Open. 2019;9: e025538. doi:10.1136/BMJOPEN-2018-025538

39. Bista B, Dhungana RR, Chalise B, Pandey AR. Prevalence and determinants of non-communicable diseases risk factors among reproductive aged women of Nepal: Results from Nepal Demographic Health Survey 2016. PLoS One. 2020;15: e0218840. doi:10.1371/JOURNAL.PONE.0218840

40. Palipudi KM, Sinha DN, Choudhury S, Zaman MM, Asma S, Andes L, et al. Predictors of tobacco smoking and smokeless tobacco use among adults in Bangladesh. Indian J Cancer. 2012;49: 387. doi:10.4103/0019-509X.107745

41. Nargis N, Thompson ME, Fong GT, Driezen P, Hussain AKMG, Ruthbah UH, et al. Prevalence and Patterns of Tobacco Use in Bangladesh from 2009 to 2012: Evidence from International Tobacco Control (ITC) Study. PLoS One. 2015;10. doi:10.1371/JOURNAL.PONE.0141135

42. Nath Khanal G, Khatri RB. Burden, prevention and control of tobacco consumption in Nepal: a narrative review of existing evidence. Int Health. 2020;0: 1–12. doi:10.1093/inthealth/ihaa055

43. Dyal SR, Valente TW. A Systematic Review of Loneliness and Smoking: Small Effects, Big Implications. https://doi.org/103109/1082608420151027933. 2015;50: 1697–1716. doi:10.3109/10826084.2015.1027933

44. Sreeramareddy CT, Pradhan PMS, Mir IA, Sin S. Smoking and smokeless tobacco use in nine South and Southeast Asian countries: Prevalence estimates and social determinants from Demographic and Health Surveys. Popul Health Metr. 2014;12: 1–16. doi:10.1186/S12963-014-0022-0/TABLES/5

45. Khoorshid Riaz B, Islam MZ, Islam S, Zaman MM, Hossain MA, Rahman MM, et al. Risk factors for non-communicable diseases in Bangladesh: findings of the population-based cross-sectional national survey 2018. BMJ Open. 2020;10: 41334. doi:10.1136/bmjopen-2020-041334

46. Khan MMH, Khan A, Kraemer A, Mori M. Prevalence and correlates of smoking among urban adult men in Bangladesh: Slum versus non-slum comparison. BMC Public Health. 2009;9: 1–12. doi:10.1186/1471-2458-9-149/TABLES/5

47. Khanam M, Osuagwu UL, Sanin KI, Haque MA, Rita RS, Agho KE, et al. Underweight, Overweight and Obesity among Reproductive Bangladeshi Women: A Nationwide Survey. Nutrients. 2021;13. doi:10.3390/NU13124408

48. Sathi NJ, Islam MA, Ahmed MS, Islam SMS. Prevalence, trends and associated factors of hypertension and diabetes mellitus in Bangladesh: Evidence from BHDS 2011 and 2017–18. PLoS One. 2022;17: e0267243. doi:10.1371/JOURNAL.PONE.0267243

49. Ali NB, Dibley MJ, Islam S, Rahman MM, Raihana S, Bhuiyan SET, et al. Overweight and obesity among urban women with iron deficiency anaemia in Bangladesh. Matern Child Nutr. 2021;17. doi:10.1111/MCN.13102

50. Afshari M, Alizadeh-Navaei R, Moosazadeh M. Oral contraceptives and hypertension in women: results of the enrolment phase of Tabari Cohort Study. BMC Womens Health. 2021;21: 1–7. doi:10.1186/S12905-021-01376-4/TABLES/4
51. Linger Endalifer M, Diress G, Addisu A, Linger B. The association between combined oral contraceptive use and overweight/ obesity: a secondary data analysis of the 2016 Ethiopia Demographic and Health Survey. BMJ Open. 2020;10: 39229. doi:10.1136/bmjopen-2020-039229
52. Abdul Baker Chowdhury M, Uddin J, Haque R, Ibrahimou B. Hypertension among adults in Bangladesh: evidence from a national cross-sectional survey. 2016. doi:10.1186/s12872-016-0197-3
53. Thang NM, Popkin BM. ORIGINAL COMMUNICATION Patterns of food consumption in Vietnam: effects on socioeconomic groups during an era of economic growth. Eur J Clin Nutr. 2004;58: 145–153. doi:10.1038/sj.ejcn.1601761
54. Al-Zubayer MA, Ahammed B, Sarder MA, Kundu S, Majumder UK, Islam SMS. Double and triple burden of non-communicable diseases and its determinants among adults in Bangladesh: Evidence from a recent demographic and health survey. Int J Clin Pract. 2021;75. doi:10.1111/IJCP.14613
55. Chowdhury S, Banu LA, Chowdhury TA, Rubayet S, Khatoon S. Achieving Millennium Development Goals 4 and 5 in Bangladesh. BJOG. 2011;118: 36–46. doi:10.1111/j.1471-0528.2011.03111.x
56. Sustainable Development Goal 3: Good Health and Well-being | United Nations in Bangladesh. [cited 1 Jul 2022]. Available: https://bangladesh.un.org/en/sdgs/3
57. Storm F, Agampodi S, Eddleston M, Sørensen JB, Konradsen F, Rheinländer T. Indirect causes of maternal death. Lancet Glob Health. 2014;2: e566. doi:10.1016/S2214-109X(14)70297-9

58. Panaitescu AM, Syngelaki A, Prodan N, Akolekar R, Nicolaides KH. Chronic hypertension and adverse pregnancy outcome: a cohort study. Ultrasound Obstet Gynecol. 2017;50: 228–235. doi:10.1002/UOG.17493

59. Anderson TM, Lavista Ferres JM, You Ren S, Moon RY, Goldstein RD, Ramirez JM, et al. Maternal Smoking Before and During Pregnancy and the Risk of Sudden Unexpected Infant Death. Pediatrics. 2019;143. doi:10.1542/PEDS.2018-3325

60. Cedergren MI. Maternal morbid obesity and the risk of adverse pregnancy outcome. Obstetrics and gynecology. 2004;103: 219–224. doi:10.1097/01.AOG.0000107291.46159.00

Table 1. Socio-demographic characteristics of participants

Variables	Un-weighted count	Weighted percent/Mean (SD)
**Total**	5,624	100.00
**Mean age (year)**		31.00 (9.07)
**Age group (year)**		
18 – 29	2,689	48.26
30 – 39	1,716	30.26
40 – 49	1,219	21.48
**Educational status**		
No education	957	17.78
Primary	1,691	29.87
Secondary	2,051	37.68
Higher	925	14.67
**Marital status**		
Never married	402	6.33
Married	4,902	88.22
Widowed/divorced	320	5.45
**Residence**		
Rural	3,533	71.84
Urban	2,091	28.16
**Division**		
Barishal	590	5.47
Chattogram	827	18.57
Dhaka	799	24.44
Khulna	729	11.69
Mymensingh	589	7.50
Rajshahi	732	14.24
Rangpur	690	11.62
Sylhet	668	6.46
**Wealth index**		
Poorest	1,069	19.01
Poorer	1,051	19.50
Middle	1,081	19.90
Richer	1,091	19.56
Richest	1,332	22.02
**Occupational status***		
Not working	3,066	54.38
Services	242	4.01
Agriculture/self-employed	1,667	30.22
Manual	641	11.39

*8 cases missing

Table 2. Prevalence of non-communicable diseases risk factors among reproductive-aged women

	Current smoking		Overweight and obesity		Hypertension	
**Variables**	**n**	**Prevalence, % (95% CI)**	**n**	**Prevalence, % (95% CI)**	**n**	**Prevalence, % (95% CI)**
**Total**	5,624	9.55 (8.65-10.53)	5,603	31.57 (30.17-33.02)	5,624	20.27 (19.11-21.49)
**Age group (year)**						
18 – 29	2,689	4.28 (3.53-5.17)	2,679	22.92 (21.11-24.82)	2,689	9.03 (7.87-10.33)
30 – 39	1,716	11.06 (9.48 -12.86)	1,708	40.26 (37.63-42.94)	1,716	24.77 (22.48-27.20)
40 – 49	1,219	19.26 (16.69 -22.12)	1,216	38.82 (35.91-41.82)	1,219	39.21 (36.16-42.35)
P-value		<0.001		<0.001		<0.001
**Educational status**						
No education	957	19.69 (17.05-22.62)	952	26.13 (23.26-29.21)	957	29.07 (25.92-32.43)
Primary	1,691	12.58 (10.95-14.41)	1,688	30.75 (28.35-33.27)	1,691	22.29 (20.16-24.57)
Secondary	2,051	5.07 (4.17-6.15)	2,044	33.81 (31.42-36.29)	2,051	17.14 (15.39-19.04)
Higher	925	2.60 (1.67-4.05)	919	34.08 (30.44-37.92)	925	13.57 (11.33-16.17)
P-value		<0.001		0.001		<0.001
**Marital status**						
Never married	402	1.39 (0.53-3.57)	396	10.43 (7.47-14.38)	402	6.18 (4.01-9.40)
Married	4,902	9.71 (8.78-10.73)	4,890	33.36 (31.88-34.87)	4,902	20.95 (19.69-22.27)
Widowed/divorced	320	16.41 (2.42-21.38)	317	26.85 (21.57-32.87)	320	25.70 (20.60-31.56)
P-value		<0.001		<0.001		<0.001
**Residence**						
Rural	3,533	10.21 (9.11-11.42)	3,519	28.20 (26.53-29.94)	3,533	19.92 (18.50-21.41)
Urban	2,091	7.87 (6.45-9.57)	2,084	40.18 (37.63-42.78)	2,091	21.19 (19.21-23.31)
P-value		0.024		<0.001		0.317
**Division**						
Barishal	590	21.29 (17.65-25.45)	588	29.28 (25.13-33.80)	590	24.24 (20.43-28.50)
Chattogram	827	8.62 (6.64-11.10)	824	36.26 (32.57-40.11)	827	23.51 (20.71-26.57)
Dhaka	799	6.35 (4.77-8.41)	795	35.73 (32.44-39.16)	799	16.90 (14.37-19.78)
Khulna	729	6.83 (4.77-9.70)	728	35.45 (31.80-39.29)	729	20.47 (17.29-24.05)
Mymensingh	589	17.01 (13.72-20.89)	587	24.69 (21.03-28.75)	589	15.76 (12.94-19.06)
Rajshahi	732	5.06 (3.24-7.82)	728	28.13 (24.64-31.90)	732	20.62 (17.36-24.32)
Rangpur	690	8.92 (6.10-12.86)	689	25.20 (21.89-28.83)	690	23.36 (20.51-26.48)
Sylhet	668	21.69 (17.73-26.24)	664	24.40 (20.11-29.26)	668	18.93 (15.78-22.55)
P-value		<0.001		<0.001		0.001
**Wealth index**						
Poorest	1,069	14.04 (11.72-16.75)	1,066	16.48 (14.10-19.19)	1,069	17.35 (14.77-20.28)
Poorer	1,051	12.46 (10.43-14.81)	1,046	21.74 (19.12-24.62)	1,051	18.84 (16.44-21.48)
Middle	1,081	8.37 (6.76-10.31)	1,080	29.80 (26.82-32.97)	1,081	20.10 (17.66-22.78)
Richer	1,091	8.42 (6.80-10.39)	1,088	34.59 (31.47-37.84)	1,091	21.39 (18.82-24.21)
Richest	1,332	5.16 (3.98-6.66)	1,323	52.31 (49.24-55.37)	1,332	23.24 (20.81-25.86)
P-value		<0.001		<0.001		0.019
**Occupational status***						
Not working	3,066	7.53 (6.53-8.67)	3,052	34.35 (32.41-36.34)	3,066	20.19 (18.65-21.83)
Services	242	5.62 (3.15-9.85)	238	35.18 (28.54-42.45)	242	20.76 (15.51-27.20)
Agriculture/self-employed	1,667	14.49 (12.57-16.63)	1,667	25.97 (23.85-28.20)	1,667	22.25 (20.03-24.63)
Manual	641	7.60 (5.33-10.72)	638	32.46 (28.52-36.68)	641	15.56 (12.83-18.75)
P-value		<0.001		<0.001		0.013

*8 cases missing

Table 3. Association between socio-demographic characteristics and non-communicable diseases risk factors identified from the multivariable mixed-effects Poisson regression model

Variables	Current smoking, APR (95% CI)	Overweight and obesity, APR (95% CI)	Hypertension, APR (95% CI)
**Age group (year)**			
18 – 29	Reference	Reference	Reference
30 – 39	1.83 (1.45-2.30)***	1.71 (1.55-1.89) ***	2.58 (2.18-3.05)***
40 – 49	2.65 (2.07-3.38)***	1.77 (1.57-1.98) ***	3.94 (3.32-4.69)***
**Educational status**			
No education	3.19 (1.85-5.49)***	0.81 (0.69-0.97)*	1.33 (1.04-1.71)*
Primary	2.41 (1.43-4.04) **	0.98 (0.85-1.12)	1.27 (1.01-1.61)*
Secondary	1.45 (0.87-2.43)	1.04 (0.92-1.17)	1.23 (0.98-1.53)
Higher	Reference	Reference	Reference
**Marital status**			
Never married	Reference	Reference	Reference
Married	2.87 (1.10-7.52)*	2.71 (1.96-3.75)***	1.60 (1.02-2.51)*
Widowed/divorced	3.32 (1.22-9.03)*	2.16 (1.49-3.15)***	1.53 (0.94-2.50)
**Residence**			
Rural	Reference	Reference	Reference
Urban	1.23 (1.00-1.50)*	1.01 (0.92-1.10)	1.06 (0.94-1.21)
**Division**			
Barishal	2.81 (2.04-3.86)***	1.07 (0.91-1.26)	1.50 (1.20-1.87)***
Chattogram	1.41 (1.00-1.98)*	1.11 (0.98-1.26)	1.45 (1.20-1.77)***
Dhaka	Reference	Reference	Reference
Khulna	0.91 (0.62-1.34)	1.11 (0.97-1.28)	1.18 (0.96-1.46)
Mymensingh	2.06 (1.47-2.90)***	0.93 (0.78-1.11)	1.02 (0.79-1.31)
Rajshahi	0.61 (0.40-0.92)*	1.01 (0.87-1.18)	1.27 (1.02-1.57)*
Rangpur	1.00 (0.68-1.46)	1.03 (0.87-1.22)	1.52 (1.23-1.89)***
Sylhet	3.07 (2.26-4.16)***	0.87 (0.74-1.02)	1.19 (0.96-1.49)
**Wealth index**			
Poorest	Reference	Reference	Reference
Poorer	1.00 (0.81-1.24)	1.27 (1.05-1.54)*	1.08 (0.89-1.31)
Middle	0.79 (0.62-1.02)	1.67 (1.39-2.01)***	1.19 (0.98-1.44)
Richer	0.90 (0.68-1.17)	1.97 (1.64-2.36)***	1.36 (1.12-1.67)**
Richest	0.66 (0.46-0.94)*	2.88 (2.39-3.47)***	1.51 (1.22-1.87)***
**Occupational status**			
Not working	Reference	Reference	Reference
Services	1.12 (0.63-2.00)	0.94 (0.77-1.14)	1.04 (0.78-1.38)
Agriculture/self-employed	1.42 (1.18 -1.71)***	0.88 (0.79-0.98)*	0.91 (0.80-1.04)
Manual	0.94 (0.69-1.27)	1.00 (0.87-1.14)	0.76 (0.62-0.93)**

APR: Adjusted prevalence ratio
***Significant at p-value < 0.001.
**Significant at p-value < 0.01.
*Significant at p-value < 0.05.

Table 4. Mean number of NCDs risk factors and multivariable analysis (mixed-effects Poisson regression model) of clustering of NCDs risk factors

Variables	Mean number (95% CI)	Clustering of NCDs risk factors, APR (95% CI)
**Age group (year)**		
18 – 29	0.36 (0.34-0.39)	Reference
30 – 39	0.76 (0.72-0.80)	1.94 (1.78-2.11)***
40 – 49	0.97 (0.92-1.03)	2.44 (2.22-2.68)***
**Educational status**		
No education	0.75 (0.69-0.80)	1.15 (1.00-1.33)*
Primary	0.66 (0.61-0.70)	1.13 (1.00-1.29)*
Secondary	0.56 (0.53-0.60)	1.09 (0.97-1.23)
Higher	0.50 (0.45-0.55)	Reference
**Marital status**		
Never married	0.18 (0.13-0.23)	Reference
Married	0.64 (0.62-0.66)	2.32 (1.78-3.04)***
Widowed/divorced	0.69 (0.59-0.78)	2.14 (1.59-2.89)***
**Residence**		
Rural	0.58 (0.56-0.61)	Reference
Urban	0.69 (0.65-0.73)	1.06 (0.99-1.14)
**Division**		
Barishal	0.75 (0.68-0.82)	1.44 (1.28-1.63)***
Chattogram	0.68 (0.63-0.74)	1.25 (1.12-1.40)***
Dhaka	0.59 (0.53-0.64)	Reference
Khulna	0.63 (0.57-0.69)	1.10 (0.97-1.24)
Mymensingh	0.58 (0.51-0.64)	1.13 (0.98-1.30)
Rajshahi	0.54 (0.48-0.60)	1.02 (0.90-1.16)
Rangpur	0.58 (0.52-0.63)	1.15 (1.01-1.32)*
Sylhet	0.65 (0.58-0.72)	1.27 (1.12-1.43)***
**Wealth index**		
Poorest	0.48 (0.43-0.53)	Reference
Poorer	0.53 (0.48-0.58)	1.11 (0.99-1.26)
Middle	0.58 (0.54-0.63)	1.25 (1.11-1.41)***
Richer	0.64 (0.59-0.69)	1.44 (1.27-1.63)***
Richest	0.81 (0.76-0.85)	1.82 (1.60-2.07)***
**Occupational status**		
Not working	0.62 (0.59-0.65)	Reference
Services	0.62 (0.50-0.73)	0.99 (0.83-1.18)
Agriculture/self-employed	0.63 (0.59-0.67)	0.98 (0.90-1.06)
Manual	0.55 (0.49-0.61)	0.90 (0.80-1.01)

APR: Adjusted prevalence ratio
***Significant at p-value < 0.001.
**Significant at p-value < 0.01.
*Significant at p-value < 0.05.

Response to Reviewers

“We are giving thanks to *the Academic Editor and the reviewer-1 and reviewer-2 for managing time to review our manuscript. The raised points have certainly improved the quality of the manuscript. We have addressed all the comments and made the required changes in our manuscript. The quality of the revised version of the manuscript is certainly improved.* ***Note: In the review report, we have directed the lines and page number of the revised manuscript with track changes****”.*

Authors’ Responses to Academic Editor:

Review Comment #1: Please ensure that you refer to Figure 1 in your text as, if accepted, production will need this reference to link the reader to the figure.

Reply from the authors:

Thank you for your comment. We have referred to Figure 1 in our text. Please, look at page number 7 and line number 167.

Authors’ Responses to Reviewer 1:

Review Comment #1: Define reproductive age group in the abstract.

Reply from the authors:

Thank you for your comment. We have defined the reproductive age group in the abstract. Please, look at page number 2 and line number 45.

Review Comment #2: The introduction section could be shortened.

Reply from the authors:

Thank you for your comment. We have deleted very few sentences, which can be found in the track changes. However, as there is no page limit in PLOS ONE, a long introduction section might not be an issue. Moreover, it is difficult to delete some sentences at this stage because all the

sentences are correlated with one another and carry an importance. Thanks for your consideration.

Review Comment #3: Line 4: Suggest replacing the word 'evidence' with 'findings' in the title considering the results are from a survey.

Reply from the authors:

Thank you for your comment. We have replaced it as you suggested.

Review Comment #4: Line 56-57: Please mention compared to which division the Barishal division had higher risk factors for NCD.

Reply from the authors:

Thank you for your comment. We have updated as you suggested. Please, look at page number 2 and lines number 57-58.

Review Comment #5: Line 81-84: The number of premature deaths reported 15 million globally in line 81, whereas in line 84 it was 38 million in year 2012. Suggest checking the number and report the most recent one incusing the year of report.

Reply from the authors:

Thank you for your comment. We have updated as you suggested. Please, look at page number 4 and lines number 81-84.

Review Comment #6: Line 85: Mention the year of data. Also report the total number of deaths.

Reply from the authors:

Thank you for your comment. We have updated as you suggested. Please, look at page number 4 and lines number 87-88.

Review Comment #7: Line 86: Report the NCD prevalence change over-time or actual prevalence during different time.

Reply from the authors:

Thank you for your comment. We have updated as you suggested. Please, look at page number 4 and lines number 88-92.

Review Comment #8: Line 97-98: Are this prevalence among total population or certain age group.

Reply from the authors:

Thank you for your comment. We have updated as you suggested. Please, look at page number 4 and line number 102.

Review Comment #9: Line 173: Check the definition used to define hypertension. The hypertension should be defined ‘SBP≥140’ OR ‘DBP≥90’ OR ‘on anti-hypertensive medication’. But in the definition provided it was ‘SBP≥140’ AND ‘DBP≥90’ OR ‘on anti-hypertensive medication’. If the later definition used to define the hypertension – redefine hypertension using correct definition and redo the analysis.

Reply from the authors:

Thank you for your comment. We used the correct definition (the first one) to define hypertension. Now, we have updated the manuscript as you suggested. It was a typo in writing. However, we conducted all our analyses based on the correct definition. We could provide our dataset and script on request.

Review Comment #10: Table 3: Include whether it was univariate or multivariate analysis.

Reply from the authors:

Thank you for your comment. We have updated as you suggested.

Review Comment #11: Figure 2: Suggest presenting participants with # of risk factors by age group or showing % for each age sub-group.

Reply from the authors:

Thank you for your comment. We have updated the figure as you suggested.

Review Comment #12: Figure 3: include the reference group and title for x-axis.

Reply from the authors:

Thank you for your comment. We have updated the figure as you suggested.

Authors' Responses to Reviewer 2:

Review Comment #1: Introduction: 1. It would be beneficial for the authors to include one or more references regarding the current NCD situation of reproductive women in LMIC.

Reply from the authors:

Thank you for your comment. In the previous version, we discussed the current NCDs situation of reproductive-aged women in LMICs. Moreover, in this revised version, we have included further information regarding the NCDs situation of reproductive-aged women in LMICs. Please, look at page number 5 and lines number 107-120.

Review Comment #2: Introduction: 2.In Page 4 line no 98, Would you kindly mention the prevalence of other NCDs?

Reply from the authors:

Thank you for your comment. We have updated as you suggested. We have mentioned the prevalence of cardiovascular diseases (CVD), metabolic syndrome, and high fasting blood glucose. Please, look at page number 5 and lines number 104-106.

Review Comment #3: The general observation is that there is need to consider language usage, thus the authors should not use colloquial language but statements should be supported by references to enhance authenticity of claims. This section can be improved.

Reply from the authors:

Thank you for your comment. We have updated as you suggested. You will find all our changes in track changes.

Review Comment #4: Methods: The methodology for the study is well explained. Perhaps two questions. 1. One would ask is that since the BDHS used a multi stage stratified sampling, how were cluster and sample design effects dealt with in the analysis of data.

Reply from the authors:

Thank you for your comment. We have updated as you suggested. However, we conducted all our analyses after accounting for the complex survey design and survey sampling weights. This time, we have included the following information in the Data Analysis section (please, look at page number 9-10 and lines number 210-211 and 223-227):

Since the BDHS survey was based on the two-stage stratified cluster sampling technique, recommended sample weights provided by the BDHS 2017-18 were used for the analyses [29]. So, to account for the complex survey design, we considered the sample weights, primary sampling units, and strata using "SVY" command of STATA.

Review Comment #5: Methods: No mention is made of the data collection instrument – was it the stepwise instrument which will allow for comparison with other studies.

Reply from the authors:

Thank you for your comment. We have updated as you suggested. This time, we have mentioned about data collection instrument in the Study Population and Data Source section (please, look at page number 7 and lines number 153-161).

Review Comment #6: Results: The result part for the study is well explained. The tables and the figures are well-documented and understandable.

Reply from the authors:

Thank you for your comment.

Review Comment #7: Discussion: This part is well written.

Reply from the authors:

Thank you for your comment.

Review Comment #8: Limitations and implementation of the study are noted and discussed.

Reply from the authors:

Thank you for your comment.
