# Supplementary material for: Prevalence and determinants of non-communicable diseases risk factors among reproductive-aged women: Findings from a nationwide survey in Bangladesh
Source: PLoS One. 2023 Jun 9;18(6):e0273128. doi: 10.1371/journal.pone.0273128 (PMC10256164; doi:10.1371/journal.pone.0273128)
Supplement: Attachment — Submitted filename: Response to Reviewers.docx [file pone.0273128.s003.docx]

**Response to Reviewers**

*“We are giving thanks* to *the Academic Editor and the reviewer-1 and reviewer-2 for managing time to review our manuscript. The raised points have certainly improved the quality of the manuscript. We have addressed all the comments and made the required changes in our manuscript. The quality of the revised version of the manuscript is certainly improved.* ***Note: In the review report, we have directed the lines and page number of the revised manuscript with track changes****”.*

**Authors’ Responses to Academic Editor:**

**Review Comment #1:** Please ensure that you refer to Figure 1 in your text as, if accepted, production will need this reference to link the reader to the figure.

**Reply from the authors:**

Thank you for your comment. We have referred to Figure 1 in our text. Please, look at page number 7 and line number 167.

**Authors’ Responses to Reviewer 1:**

**Review Comment #1:**  Define reproductive age group in the abstract.

**Reply from the authors:**

Thank you for your comment. We have defined the reproductive age group in the abstract. Please, look at page number 2 and line number 45.

**Review Comment #2:**  The introduction section could be shortened.

**Reply from the authors:**

Thank you for your comment. We have deleted very few sentences, which can be found in the track changes. However, as there is no page limit in PLOS ONE, a long introduction section might not be an issue. Moreover, it is difficult to delete some sentences at this stage because all the sentences are correlated with one another and carry an importance. Thanks for your consideration.

**Review Comment #3:**  Line 4: Suggest replacing the word ‘evidence’ with ‘findings’ in the title considering the results are from a survey.

**Reply from the authors:**

Thank you for your comment. We have replaced it as you suggested.

**Review Comment #4:**  Line 56-57: Please mention compared to which division the Barishal division had higher risk factors for NCD.

**Reply from the authors:**

Thank you for your comment. We have updated as you suggested. Please, look at page number 2 and lines number 57-58.

**Review Comment #5:**  Line 81-84: The number of premature deaths reported 15 million globally in line 81, whereas in line 84 it was 38 million in year 2012. Suggest checking the number and report the most recent one incusing the year of report.

**Reply from the authors:**

Thank you for your comment. We have updated as you suggested. Please, look at page number 4 and lines number 81-84.

**Review Comment #6:**  Line 85: Mention the year of data. Also report the total number of deaths.

**Reply from the authors:**

Thank you for your comment. We have updated as you suggested. Please, look at page number 4 and lines number 87-88.

**Review Comment #7:**  Line 86: Report the NCD prevalence change over-time or actual prevalence during different time.

**Reply from the authors:**

Thank you for your comment. We have updated as you suggested. Please, look at page number 4 and lines number 88-92.

**Review Comment #8:**  Line 97-98: Are this prevalence among total population or certain age group.

**Reply from the authors:**

Thank you for your comment. We have updated as you suggested. Please, look at page number 4 and line number 102.

**Review Comment #9:**  Line 173: Check the definition used to define hypertension. The hypertension should be defined ‘SBP≥140’ OR ‘DBP≥90’ OR ‘on anti-hypertensive medication’. But in the definition provided it was ‘SBP≥140’ AND ‘DBP≥90’ OR ‘on anti-hypertensive medication’. If the later definition used to define the hypertension – redefine hypertension using correct definition and redo the analysis.

**Reply from the authors:**

Thank you for your comment. We used the correct definition (the first one) to define hypertension. Now, we have updated the manuscript as you suggested. It was a typo in writing. However, we conducted all our analyses based on the correct definition. We could provide our dataset and script on request.

**Review Comment #10:**  Table 3: Include whether it was univariate or multivariate analysis.

**Reply from the authors:**

Thank you for your comment. We have updated as you suggested.

**Review Comment #11:**  Figure 2: Suggest presenting participants with # of risk factors by age group or showing % for each age sub-group.

**Reply from the authors:**

Thank you for your comment. We have updated the figure as you suggested.

**Review Comment #12:**  Figure 3: include the reference group and title for x-axis.

**Reply from the authors:**

Thank you for your comment. We have updated the figure as you suggested.

**Authors’ Responses to Reviewer 2:**

**Review Comment #1:**  Introduction: 1. It would be beneficial for the authors to include one or more references regarding the current NCD situation of reproductive women in LMIC.

**Reply from the authors:**

Thank you for your comment. In the previous version, we discussed the current NCDs situation of reproductive-aged women in LMICs. Moreover, in this revised version, we have included further information regarding the NCDs situation of reproductive-aged women in LMICs. Please, look at page number 5 and lines number 107-120.

**Review Comment #2:**  Introduction: 2.In Page 4 line no 98, Would you kindly mention the prevalence of other NCDs?

**Reply from the authors:**

Thank you for your comment. We have updated as you suggested. We have mentioned the prevalence of cardiovascular diseases (CVD), metabolic syndrome, and high fasting blood glucose. Please, look at page number 5 and lines number 104-106.

**Review Comment #3:** The general observation is that there is need to consider language usage, thus the authors should not use colloquial language but statements should be supported by references to enhance authenticity of claims. This section can be improved.

**Reply from the authors:**

Thank you for your comment. We have updated as you suggested. You will find all our changes in track changes.

**Review Comment #4:** Methods: The methodology for the study is well explained. Perhaps two questions. 1. One would ask is that since the BDHS used a multi stage stratified sampling, how were cluster and sample design effects dealt with in the analysis of data.

**Reply from the authors:**

Thank you for your comment. We have updated as you suggested. However, we conducted all our analyses after accounting for the complex survey design and survey sampling weights. This time, we have included the following information in the Data Analysis section (please, look at page number 9-10 and lines number 210-211 and 223-227):

Since the BDHS survey was based on the two-stage stratified cluster sampling technique, recommended sample weights provided by the BDHS 2017-18 were used for the analyses [29]. So, to account for the complex survey design, we considered the sample weights, primary sampling units, and strata using “SVY” command of STATA.

**Review Comment #5:** Methods: No mention is made of the data collection instrument – was it the stepwise instrument which will allow for comparison with other studies.

**Reply from the authors:**

Thank you for your comment. We have updated as you suggested. This time, we have mentioned about data collection instrument in the Study Population and Data Source section (please, look at page number 7 and lines number 153-161).

**Review Comment #6:**  Results: The result part for the study is well explained. The tables and the figures are well-documented and understandable.

**Reply from the authors:**

Thank you for your comment.

**Review Comment #7:**  Discussion: This part is well written.

**Reply from the authors:**

Thank you for your comment.

**Review Comment #8:**  Limitations and implementation of the study are noted and discussed.

**Reply from the authors:**

Thank you for your comment.
