# Supplementary material for: Prevalence and determinants of non-communicable diseases risk factors among reproductive-aged women: Findings from a nationwide survey in Bangladesh
Source: PLoS One. 2023 Jun 9;18(6):e0273128. doi: 10.1371/journal.pone.0273128 (PMC10256164; doi:10.1371/journal.pone.0273128)
Supplement: Attachment — Submitted filename: Response to Academic Editor and Reviewers_Feb25_2023.docx [file pone.0273128.s005.docx]

**Response to Academic Editor and Reviewers**

*Dear Dr. A. K. M. Anisur Rahman,*

*Academic Editor*

*PLOS ONE*

*We thank you for managing the time to review and handle this manuscript. We submitted our revised version-1 addressing all the comments from reviewer-1 and reviewer-2. Now, we have received feedback from reviewer-3 and reviewer-4, which is usually not a regular instance in PLOS ONE. However, we appreciate this technique as this critical appraisal will certainly help to improve the quality of the manuscript, and we are delighted to respond to all the comments raised by the academic editor and reviewers.*

*We are also giving thanks* *to* *reviewer-3 and reviewer-4 for managing time to review our manuscript. The raised points have certainly improved the quality of the manuscript. We have addressed all the comments and made the required changes in our manuscript. The quality of the revised version of the manuscript is certainly improved.* ***Note: In the review report, we have directed the lines and page number of the revised manuscript with track changes****.*

*Many thanks for considering.*

*Warm regards,*

*Saifur Rahman Chowdhury, MPH (Epidemiology)*

*PhD Student (Clinical Epidemiology)*

*Department of Health Research Methods, Evidence, and Impact (HEI), McMaster University, Hamilton, Ontario, Canada*

**Authors’ Responses to Academic Editor:**

**Comment #1:** Smoking, highpertension, overweight and obesity are binary outcome variables. How did the authors conduct their Poisson regression analysis with binary outcome variables? Did they count the number of women in households, unions, upazilas, and districts who had NCD risk factors before performing an analysis?

**Reply from the authors:**

Thank you for your comment.

We did not use the conventional Poisson regression model. We used a modified Poisson regression model with robust error variance. The modified Poisson regression model with a robust error variance is used as an alternative logistic regression model if the prevalence of binary outcome is common (i.e., more than >10%). We used this model since the odds ratio estimated using logistic regression from a cross-sectional study may significantly overestimate relative risk when the outcome is common **(Ref-1 has got >4,000 citations)** [1,2]. Secondly, in the case of convergence failure with the log-binomial model, modified Poisson regression with a robust error variance performs better in estimating the prevalence ratio from a cross-sectional study **(Ref-4 has got >7,000 citations)** [3,4]. Many studies utilized this modified Poisson regression model, which have been recently published in reputed journals [5–7]. We have explained this process in the data analysis section also.

Moreover, for the cluster of NCDs risk factors, we performed Poisson regression model with robust error variance for the count data. The number of risk factors from smoking, overweight/obesity, or hypertension was counted to assess the clustering of risk factors. Therefore, their existence is deemed to range from 0 to 3. We used the robust error variance option to obtain robust standard errors for the parameter estimates as recommended by Cameron and Trivedi (2009) to control for mild violations of underlying assumptions [8].

1.	**Barros AJD, Hirakata VN. Alternatives for logistic regression in cross-sectional studies: An empirical comparison of models that directly estimate the prevalence ratio. BMC Med Res Methodol. 2003;3: 1–13. doi:10.1186/1471-2288-3-21/TABLES/9**

2.	Tamhane AR, Westfall AO, Burkholder GA, Cutter GR. Prevalence odds ratio versus prevalence ratio: choice comes with consequences. Stat Med. 2016;35: 5730–5735. doi:10.1002/SIM.7059

3.	Yelland LN, Salter AB, Ryan P. Performance of the Modified Poisson Regression Approach for Estimating Relative Risks From Clustered Prospective Data. Am J Epidemiol. 2011;174: 984–992. doi:10.1093/AJE/KWR183

4.	**Zou G. A modified poisson regression approach to prospective studies with binary data. Am J Epidemiol. 2004;159: 702–706. doi:10.1093/AJE/KWH090**

5.	Huda MN, Billah M, Sharmin S, Amanullah ASM, Hossin MZ. Associations between family social circumstances and psychological distress among the university students of Bangladesh: To what extent do the lifestyle factors mediate? BMC Psychol. 2021;9. doi:10.1186/S40359-021-00587-6

6.	Rana J, Islam RM, Khan MN, Aliani R, Oulhote Y. Association between household air pollution and child mortality in Myanmar using a multilevel mixed-effects Poisson regression with robust variance. Sci Rep. 2021;11. doi:10.1038/s41598-021-92193-0

7.	Rana J, Uddin J, Peltier R, Oulhote Y. Associations between Indoor Air Pollution and Acute Respiratory Infections among Under-Five Children in Afghanistan: Do SES and Sex Matter? Int J Environ Res Public Health. 2019;16. doi:10.3390/IJERPH16162910

8.	Cameron AC, Trivedi PK. Microeconometrics Using Stata, Second Edition. Stata Press; 2022.

**Comment #2:** As the authors described, the terms "overweight" and "obesity" have different definitions. However, they combined the two to form a single outcome variable. How is that even possible?

**Reply from the authors:**

Thank you for your comment.

The overweight was considered for the individual with BMI between 25.0 and 29.9 kg/m^2^, and the obese was BMI greater than or equal to 30.0 kg/m^2^. We combined overweight and obese to make a single binary variable and defined an individual in the overweight/obese category if the BMI was greater than or equal to 25.0 kg/m^2^.

**Comment #3:** The authors analyzed the data in four different ways to identify the determinants for NCD risk factors: three for three risk factors and one overall. However, the results were presented only for overall determinants in the abstract.

**Reply from the authors:**

Thank you for your comment.

Yes, we individually analyzed data for three different risk factors (outcome variables) using the same independent variables. Finally, the number of risk factors present within each participant (from 0 to 3) was counted to assess the clustering of risk factors and analyzed using the Poisson regression model with robust error variance. We have updated our abstract and given the information about all three specific outcome variables (smoking, overweight/obesity, and hypertension) and the cluster of NCDs risk factors.

**Comment #4:** The authors presented multivariable Poisson regression model results without presenting the corresponding univariable screening results and the criteria for variables to be included in the multivariable models.

**Reply from the authors:**

Thank you for your comment.

Differences between categorical variables were tested using the chi-square tests. We included variables in the multivariable models if the chi-square test p-value <0.20 or based on their clinical relevance. We have added this information in the data analysis section also.

**Authors’ Responses to Reviewer 1:**

**Review Comment:**  All the review comments were given in the pdf of the submitted file.

**Reply from the authors:**

Thank you for your comment. We have taken reviewer’s comment attentively and addressed and corrected accordingly. All the changes can be found in the track changes. Moreover, we responded in the pdf where the reviewer commented. The file is attached.

**Authors’ Responses to Reviewer 2:**

**Review Comment #1:**  Many countries have done and are doing NCD RF surveys as a standalone or as a part of the DHS surveys. Beyond a point there is little usefulness of prevalence and predictors of the risk factors. These are no longer research questions but more of surveillance and monitoring for change. The paper does not really add any valuable knowledge and is at best a confirmation of what is known.

**Reply from the authors:**

Thank you for your insightful comment. We acknowledge that these types of studies have been conducted before in different countries. However, women in the reproductive age group are a significant portion of a country's population. In the context of Bangladesh, no studies have been conducted before focusing on this important group of the population of the country. A few related studies were conducted before using earlier data. Here, we have used the most recent DHS data presenting the updated information on the research question. So, the findings from this study might be instrumental in drawing the attention of the respective authorities and policymakers of the country. Studies can always be repeated, and all studies give some unique information and add some new knowledge. This is the importance of a study.

**Review Comment #2:**  DHS is largely an platform for RCH monitoring and and has added three variables related to NCDs and focuses only among women.

**Reply from the authors:**

Thank you for your comment. BDHS is the only available nationwide database (survey) that has only three NCDs risk factors (hypertension, overweight/obesity, smoking). There is no other nationwide data source in Bangladesh to include more NCDs risk factors, so we could not think apart from this. Moreover, these are the most common NCDs risk factors. We focused on the reproductive age group women as this age group is largely significant.

**Review Comment #3:** It would be better if a section on trends is added with maybe a table or diagram to make the paper more relevant.

**Reply from the authors:**

Thank you for your insightful comment. This could be another research question, and we are planning to work on another paper addressing the trends and changes.

**Review Comment #4:** Since only three risk factors are considered, clustering in this does not match the other NCDRF surveys. Clustering is not clearly defined in the paper.

**Reply from the authors:**

Thank you for your comment. Here, we have defined clustering as the cumulative number of risk factors in an individual. We assumed that it is important to identify which group of people are being experienced with higher/multiple NCDs risk factors. We have added a section in the methods section (outcome variable) defining the clustering of NCDs risk factors. Now it is much clearer than before. This section is also given below:

**Clustering of NCDs risk factors:** The clustering of NCDs risk factors was defined as the existence of multiple risk factors in each participant. The number of risk factors from smoking, overweight/obesity, or hypertension was counted to assess the clustering of risk factors. Therefore, their existence is deemed to range from 0 to 3.

**Review Comment #5:** Introduction: The para on global women status can be deleted or shortened. Only those issues covered in the paper to be retained.

**Reply from the authors:**

Thank you for your comment. We have updated as you suggested. The changes can be found in the track changes.

**Review Comment #6:**  Drop table 1 (as details are there in table 2) and figure 1 (not specific to this study).

**Reply from the authors:**

Thank you for your comment. In Table 1, we presented the frequency and percentage of each variable and category. However, in Table 2, we presented the prevalence of smoking, overweight/obesity, and hypertension according to different variables. Both tables represent different information. So, the authors feel that keeping both tables will make it more reader-friendly.

Figure 1 represents the data collection process of the Bangladesh Demographic and Health Survey and, finally, how we selected our analytic sample. Most of the articles published using DHS data use similar figures. We have made modifications to the figure, and we have given the legend accordingly. So, the authors feel that the figure could be helpful for the reader to understand the study sample.

**Review Comment #7:**  Table 2 can delete one NU column by getting Hypertension column before obesity as the numbers are the same.

**Reply from the authors:**

We highly appreciate your suggestion. However, presenting in the order of smoking, overweight/obesity, and hypertension makes more sense. Moreover, giving number column for two outcome variables and missing it for one outcome variable will make it ambiguous. So, upon respecting the reviewer’s suggestion, the authors feel that keeping the table in its current form is more reader-friendly.

**Review Comment #8:**  Limitation of causality is unwarranted as no one imputes causality in a cross-sectional study and that was never the intent of the survey.

**Reply from the authors:**

Thank you for your super helpful comment. We have removed it and updated accordingly.
