# Supplementary material for: Prevalence and determinants of non-communicable diseases risk factors among reproductive-aged women: Findings from a nationwide survey in Bangladesh
Source: PLoS One. 2023 Jun 9;18(6):e0273128. doi: 10.1371/journal.pone.0273128 (PMC10256164; doi:10.1371/journal.pone.0273128)
Supplement: Attachment — Submitted filename: Reviewer comments.docx [file pone.0273128.s002.docx]

**Revision of the study “Prevalence and determinants of non-communicable diseases risk factors among reproductive-aged women of Bangladesh: Evidence from Bangladesh Demographic Health Survey 2017-2018”**

This study aims to investigate the Prevalence and determinants of non-communicable diseases risk factors among reproductive-aged women of Bangladesh: Evidence from Bangladesh Demographic Health Survey 2017-2018.

I have listed some specific comments below that the authors should consider before this work could be published.

**Introduction:**

1. It would be beneficial for the authors to include one or more references regarding the current NCD situation of reproductive women in LMIC.

2.In Page 4 line no 98, Would you kindly mention the prevalence of other NCDs?

The general observation is that there is need to consider language usage, thus the authors should not use colloquial language but statements should be supported by references to enhance authenticity of claims. This section can be improved.

**Methods:**

The methodology for the study is well explained. Perhaps two questions

1. One would ask is that since the BDHS used a multi stage stratified sampling, how were cluster and sample design effects dealt with in the analysis of data**.**

**2.** No mention is made of the data collection instrument – was it the stepwise instrument which will allow for comparison with other studies.

**Results:**

The result part for the study is well explained. The tables and the figures are well-documented and understandable.

**Discussion:**

This part is well written.

Limitations and implementation of the study are noted and discussed.
